# Species‐level controls of foliar methane and nitrous oxide fluxes: roles of traits and microbes in temperate trees

**DOI:** 10.1111/nph.71433

**Published:** 2026-07-13

**Authors:** Md Rezaul Karim, Sean C. Thomas

**Affiliations:** ^1^ Institute of Forestry and Conservation, John H Daniels Faculty of Architecture Landscape and Design University of Toronto 33 Willcocks St. Toronto M5S 3B3 ON Canada

**Keywords:** foliar gas exchange, GWP, methane uptake, methanotrophs, nitrous oxide emission, temperate forest

## Abstract

- The contribution of tree foliage to atmospheric methane (CH_4_) and nitrous oxide (N_2_O) fluxes remains uncertain in global greenhouse gas (GHG) budgets. We investigated whether species identity, shade tolerance, and leaf‐associated microbes influence foliar CH_4_ and N_2_O exchange.
- We conducted repeated *in situ* measurements of foliar CH_4_ and N_2_O fluxes across 25 temperate tree species interplanted at a forest restoration site using high‐resolution laser spectroscopy, complemented by colocated soil flux measurements, and targeted microbial DNA sequencing.
- Tree foliage consistently acted as a net CH_4_ sink and net N_2_O source. Foliar CH_4_ oxidation increased by *c.* 33% in fall and was approximately threefold higher in shade‐tolerant than shade‐intolerant angiosperms, whereas N_2_O emissions were highest in shade‐intolerant species with limited seasonality. Species identity explained most flux variability, with the highest‐CH_4_‐oxidizing angiosperm (*Tilia americana*) showing an order‐of‐magnitude higher particulate methane monooxygenase (*pmoA*) copy numbers and much greater Type I methanotroph representation than the lowest‐CH_4_‐oxidizing species (*Prunus virginiana*).
- Foliar CH_4_ uptake dominated the net non‐CO_2_ climate forcing effect despite consistent N_2_O emissions. These findings highlight tree species identity and shade tolerance as key axes of foliar CH_4_ uptake and N_2_O emission, with abundance and species composition of leaf‐associated methanotrophs also tracking species‐level variation in foliar CH_4_ uptake.

## Graphical abstract

Species‐level variation in temperate tree foliar greenhouse gas exchange, showing consistent foliar CH_4_ uptake, N_2_O emission, stronger CH_4_ uptake in fall leaves, and contrasting methanotroph communities between the high‐CH_4_‐oxidizing species *Tilia americana* and the low‐CH_4_‐oxidizing species *Prunus virginiana*.

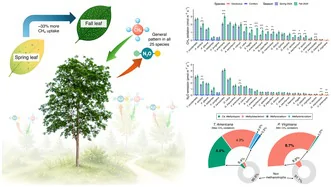

## Introduction

Methane (CH_4_) and nitrous oxide (N_2_O) are highly potent biogenic greenhouse gases (GHGs) with global warming potentials 28–34 and 265–298 times greater than CO_2_, respectively, over a 100‐yr timescale (Pachauri *et al*., 2014; Nisbet *et al*., 2021). Since the preindustrial period, atmospheric concentrations of CH_4_ and N_2_O have increased by *c*. 156% and 23%, respectively, resulting in estimated radiative forcings of 0.54 W m^−2^ (CH_4_) and 0.21 W m^−2^ (N_2_O) as reported in the IPCC Sixth Assessment Report 2021 (Masson‐Delmotte *et al*., 2021). While anthropogenic sources such as agriculture and fossil fuel combustion dominate global budgets, terrestrial ecosystems – including soils and vegetation – represent significant regulators of CH_4_ and N_2_O fluxes. Mechanisms of aboveground exchange, particularly via foliage, remain poorly constrained, limiting their representation in climate models.

Historically, CH_4_ and N_2_O research has focused on soil and wetland systems, in which anaerobic and redox‐sensitive microbial processes mediate most fluxes (Aurangojeb *et al*., 2017; Pangala *et al*., 2017; Covey & Megonigal, 2019). However, recent studies show that trees, including stems and leaves, can act as both sources and sinks of CH_4_ and N_2_O (Barba *et al*., 2019; Gorgolewski *et al*., 2022, 2023; Karim *et al*., 2024). Although per‐leaf‐area fluxes are low, the global foliage surface area exceeds 1.5 × 10^8^ km^2^ (Asner *et al*., 2003), suggesting that even modest fluxes may have substantial ecosystem‐level impacts (Gauci *et al*., 2025).

Early interest in foliar CH_4_ exchange arose from a report of aerobic CH_4_ emissions from plants by Keppler *et al*. (2006); however, this result was challenged due to methodological artefacts, with studies suggesting that UV‐induced photochemical reactions contribute to only very small CH_4_ fluxes *in situ* (Kirschbaum *et al*., 2006; Dueck *et al*., 2007). Current evidence indicates that xylem‐mediated transport of soil‐derived CH_4_ and internal microbial oxidation are the main mechanisms determining foliar CH_4_ exchange (Putkinen *et al*., 2021; Gorgolewski *et al*., 2023; Karim *et al*., 2025). In parallel, phyllosphere microbial communities have been shown to include methanotrophic bacteria, particularly Type II taxa such as *Methylocystis* and *Methylosinus*, which colonize leaves and are capable of oxidizing CH_4_ under well‐lit, oxic conditions (Iguchi *et al*., 2012, 2013). However, while marker gene detection (e.g. *pmoA*) supports their presence, direct evidence of active CH_4_ oxidation remains scarce, and the functional role of phylloplane microbes vs endophytes is still unresolved.

Similarly, foliar N_2_O exchange has only recently been recognized as a potentially important flux pathway. Emissions typically dominate over uptake (Karim *et al*., 2024, 2025). Translocation of soil‐derived N_2_O via xylem flow has been demonstrated (Welch *et al*., 2019), but there is also compelling evidence for endogenous N_2_O production through internal plant processes. One pathway involves mitochondrial reduction of nitric oxide (NO) to N_2_O under hypoxic stress, serving as a detoxification mechanism (Timilsina *et al*., 2020). Recent surface sterilization experiments have shown no significant difference in foliar N_2_O emission between sterilized and nonsterilized leaves (Qin *et al*., 2024), suggesting that microbial surface processes are not an important source. Nevertheless, the relative contributions of soil‐derived transport, internal plant metabolism, and possible microbial pathways remain difficult to separate in field studies. Physiological parameters such as foliar nitrogen content, stomatal conductance, and transpiration rates are likely to regulate these pathways (Karim *et al*., 2025) but remain poorly quantified across species.

Despite increasing recognition of foliar contributions to forest GHG budgets, *in situ* survey studies remain limited in number, geographic scope, and taxonomic breadth (Gorgolewski *et al*., 2023; Karim *et al*., 2025). Existing studies have largely focused on tropical or wetland systems (Pangala *et al*., 2017; Karim *et al*., 2024), with temperate forests – particularly broadleaf and needleleaf tree species – receiving minimal attention. Available data are restricted to a small number of species, leaving significant gaps in our understanding of interspecific and functional trait‐based variability in foliar fluxes.

Methodological limitations have also constrained progress. Early studies frequently employed static chamber plus gas chromatography techniques, which are inadequate for resolving low‐level foliar gas fluxes and are prone to artifacts under fluctuating environmental conditions (Takahashi *et al*., 2012). Recent advances in dynamic chamber systems coupled with laser‐based spectroscopy (e.g. Cavity Ring‐Down Spectroscopy, off‐axis integrated cavity output spectroscopy (OA‐ICOS), Tunable Infrared Laser Differential Absorption Spectroscopy) now permit rapid sub‐ppb measurements of CH_4_ and N_2_O under field conditions, enabling continuous, high‐resolution flux estimates (Barba *et al*., 2019; Gorgolewski *et al*., 2023; Karim *et al*., 2024). Additionally, these systems allow analyses of temporal variation in foliar CH_4_ and N_2_O exchange – linked to diel cycles, phenological stage, and environmental conditions such as light and water availability (Kohl *et al*., 2023; Tenhovirta *et al*., 2024; Karim *et al*., 2025).

To date, microbial studies related to foliar CH_4_ exchange have relied on marker genes, such as *pmoA*, without assessing transcription, localization, or activity (Moisan *et al*., 2025), leaving the functional role of foliar methanotrophs unresolved. Existing metagenomic and marker‐gene surveys are rarely integrated with field‐based CH_4_ flux measurements; thus, microbial presence indicates potential rather than providing direct evidence of function. The potential role of methanotrophic endophytes provides a framework to predict seasonal and interspecific variability in CH_4_ oxidation. If methanotroph colonization or proliferation progresses through the growing season, CH_4_ oxidation rates should increase from spring to fall as endophytic communities mature. This pattern is likely to be most pronounced in deciduous species, with annually developing leaves, and less so in evergreen conifers, in which needles persist for multiple years and host stable microbial assemblages (Baldrian, 2017). If methanotroph abundance drives oxidation capacity, as observed in soils (Lima *et al*., 2014) and bark (Jeffrey *et al*., 2021), species with higher foliar CH_4_ uptake should exhibit greater representation of methanotrophic taxa.

In addition to microbial influences, functional traits related to shade tolerance and successional status may predict interspecific variation in foliar CH_4_ and N_2_O exchange. Shade‐tolerant, late‐successional species maintain longer leaf lifespans (Seiwa *et al*., 2006), potentially facilitating progressive colonization by methanotrophic endophytes. Although leaf lifespan of temperate‐zone trees is limited by growing season length, temperate pioneer species undergo multiple leaf flushes and retain foliage for shorter periods (Kikuzawa, 2003), potentially limiting microbial establishment. Lower water fluxes in shade‐tolerant species (Zhang *et al*., 2016) may also reduce xylem transport of soil‐derived CH_4_, enhancing the relative role of *in situ* oxidation. Higher foliar nitrogen in pioneer species may increase endogenous N_2_O production, explaining lower CH_4_ oxidation and higher N_2_O emissions, consistent with tropical forest observations (Karim *et al*., 2024). These considerations guided this study, in which we surveyed diverse temperate trees to identify variability in foliar CH_4_ oxidation and examine links with tree shade tolerance, seasonality, and microbial composition. Because CH_4_ oxidation is directly linked to methanotrophic taxa, microbial characterization focused on CH_4_ rather than N_2_O.

Here, we test two linked hypotheses. First, we hypothesize that foliar CH_4_ and N_2_O fluxes will vary among species and seasons, with part of this variation organized by shade tolerance/successional strategy. Specifically, we expect CH_4_ oxidation: to be greater in shade‐tolerant species, particularly among deciduous angiosperms; and to increase from spring to fall as foliage matures. We also expect N_2_O emissions to vary among species and to be associated with foliar transpiration, soil N_2_O flux, soil nitrogen, and foliar nitrogen. Second, we hypothesize that species with contrasting CH_4_ uptake will differ in the representation of leaf‐associated methanotrophic taxa. By integrating *in situ* flux measurements with shade tolerance classifications, selected leaf and soil parameters, and focused microbial characterization for CH_4_, this study provides a species‐level assessment of foliar CH_4_ and N_2_O exchange in temperate trees and identifies ecological axes that may help explain and predict variation in these fluxes.

## Materials and Methods

### Description of the study area

Field measurements were conducted at Downsview Park, a *c*. 261‐ha urban green space in north‐central Toronto, Ontario (43.7437°N, 79.4831°W), located within the Humber River–Black Creek watershed and managed by Canada Lands Co. (Supporting Information Fig. S1), within the Mixedwood Plains Ecozone (Genco, 2007; Crins *et al*., 2009). Our study focused on a young mixed‐species forest restoration site, planted in 2017–2018 with spatially interspersed species (Fig. S1); sample trees were thus *c*. 6–7 yr post‐planting at the time of measurement. The site sits on gently undulating glacial until at 185‐ to 200‐m elevation (Saurette *et al*., 2021) and generally low nutrients (Karim *et al*., 2025), originally underlain by Jeddo clay loam, a poorly drained gleysol formed from glacial deposits (Saurette *et al*., 2021), since altered by development into human‐mixed and amended surface layers (De Sousa & Spiess, 2018). Under the World Reference Base for Soil Resources, these soils are classified as ‘transportic’ or ‘anthropic’ (FAO, 2014; Burghardt *et al*., 2015); in the USDA system, they fall under Human‐Altered or Human‐Transported or Human‐Altered Material (Galbraith, 2018). Bulk density (*c*. 1.22 g cm^−3^) falls within the range typical of urban industrial and parkland soils (Pouyat *et al*., 2007).

The regional climate is humid continental (Köppen Dfb), with a mean annual temperature of *c*. 9.7°C and mean annual precipitation of *c*. 822 mm (Environment Canada, 2025). The herbaceous layer includes naturalized and native species including *Solidago canadensis* L. (Canadian goldenrod) and *Poa pratensis* L. (Kentucky bluegrass) (Downsview Park, 2025). Collectively, the site represents a multifunctional urban forest ecotone.

### Species selection

A total of 25 tree species were selected for foliar gas‐exchange measurements within the study site (Table 1). Species selection was based on two criteria: (1) accessibility of foliage within the 1.0–1.5 m aboveground height, suited for chamber‐based sampling; and (2) representation across major functional groups: deciduous broadleaf (*n* = 20) and coniferous needleleaf (*n* = 5) taxa. Each species was replicated with five individual trees (125 tree total). Species‐specific tolerance values to shade, drought, and waterlogging, reflecting key functional attributes potentially influencing foliar GHG exchange, were compiled from Niinemets & Valladares (2006). This globally harmonized five‐level scale (1 = very intolerant to 5 = very tolerant), constructed through cross‐calibration of continental datasets for 806 temperate Northern Hemisphere woody species, using physiological and ecological evidence of growth under limiting light and water conditions. Shade tolerance was treated as the primary ecological grouping variable because it reflects species‐level capacity for growth under low‐light conditions and is commonly associated with successional strategy and resource‐use ecology in temperate trees. It was not used as a direct measure of the light environment, canopy position, or leaf lifespan – foliage was sampled from accessible lower‐canopy leaves and measured under standardized chamber irradiance. Drought and waterlogging tolerance, along with measured leaf and soil variables, were evaluated as supporting explanatory variables rather than as components of a full multivariate trait framework.

**Table 1:** Species included in this study.

Species name	Scientific name	Family	Types	Shade tolerance class
Eastern white pine	*Pinus strobus* L.	Pinaceae	Conifers	3
Red twig dogwood	*Cornus sericea* L.	Cornaceae	Deciduous	3
Staghorn Sumac	*Rhus typhina* L.	Anacardiaceae	Deciduous	2
White ash	*Fraxinus americana* L.	Oleaceae	Deciduous	2
American elm	*Ulmus americana* L.	Ulmaceae	Deciduous	3
White spruce	*Picea glauca* (Moench) Voss	Pinaceae	Conifers	4
Chokecherry	*Prunus virginiana* L.	Rosaceae	Deciduous	3
Pin oak	*Quercus palustris* Münchh.	Fagaceae	Deciduous	2
Eastern white cedar	*Thuja occidentalis* L.	Cupressaceae	Conifers	3
Sugar maple	*Acer saccharum* Marshall	Sapindaceae	Deciduous	5
Guelder rose	*Viburnum opulus* L.	Adoxaceae	Deciduous	3
Northern red oak	*Quercus rubra* L.	Fagaceae	Deciduous	3
Red maple	*Acer rubrum* L.	Sapindaceae	Deciduous	3
Kentucky coffee tree	*Gymnocladus dioicus* (L.) K. Koch	Fabaceae	Deciduous	3
Silver maple	*Acer saccharinum* L.	Sapindaceae	Deciduous	4
Paper birch	*Betula papyrifera* Marshall	Betulaceae	Deciduous	2
Balsam fir	*Abies balsamea* (L.) Mill.	Pinaceae	Conifers	5
Freeman's maple	*Acer × freemanii* A.E. Murray	Sapindaceae	Deciduous	4
Trembling aspen	*Populus tremuloides* Michx.	Salicaceae	Deciduous	1
Bur oak	*Quercus macrocarpa* Michx.	Fagaceae	Deciduous	3
Crack willow	*Salix × fragilis* L.	Salicaceae	Deciduous	1
Honey locust	*Gleditsia triacanthos* L.	Fabaceae	Deciduous	2
Basswood	*Tilia americana* L.	Malvaceae	Deciduous	4
Northern catalpa	*Catalpa speciosa* (Warder) Warder ex Engelm	Bignoniaceae	Deciduous	2
Blue spruce	*Picea pungens* Engelm.	Pinaceae	Conifers	4

Scientific names were verified and standardized using the National Center for Biotechnology Information (NCBI) Taxonomy database (Schoch *et al*., 2020). Species‐specific shade‐tolerance values were compiled from Niinemets & Valladares (2006).

### Data collection

Our sampling strategy was designed to maximize both taxonomic breadth and within‐species replication. Measurements were conducted during two seasonal periods – early spring (mid‐May 2024) and late fall (mid‐October 2024) – to capture phenological extremes in leaf development. The spring campaign coincided with new leaf in deciduous broadleaf species, while the fall campaign targeted fully mature or senescing foliage. The same five tagged trees per species were measured in both seasons: 125 trees across 25 species (20 deciduous broadleaf, 5 coniferous needleleaf), spanning 15 angiosperm and gymnosperm families. In each season, two leaves were selected per tagged tree for gas‐exchange measurements, giving 10 leaf measurements per species and 250 per season. As both leaves came from the same tree, they were treated as within‐tree subsamples rather than independent tree‐level replicates.

Trees were selected from upland planting locations – local areas without visible waterlogging or standing water, with different species spatially interspersed. All sampled trees had a diameter at breast height (DBH) > 1 cm (mean ± SD: 10.7 ± 4.2 cm). Gas‐exchange measurements targeted recently fully expanded foliage (Thomas & Bazzaz, 1999), with two leaves measured per individual across five replicate trees per species (*n* = 10 leaves per species per season). All measurements used a full‐spectrum photodiode chamber light source delivering 1000 μmol m^−2^ s^−1^ photosynthetic photon flux density (PPFD), representative of natural mid‐day irradiance in sun‐acclimated leaves. Leaves were acclimated to this irradiance for at least 5 min before reaching steady‐state gas exchange. Each foliar measurement consisted of three closed‐dynamic loops of 60 s each, interspersed with 60‐s ambient‐air flushing phases, and regulated by an automated valve system (Fig. S2).

We employed an integrated automated system: an OA‐ICOS analyzer for CO_2_, CH_4_, and H_2_O (LGR 915‐0011; Los Gatos Research, CA, USA) and an optical feedback cavity‐enhanced absorption spectroscopy analyzer for N_2_O (LI‐7820; LI‐COR Inc., Lincoln, NE, USA). This setup is validated for detecting low‐level CH_4_ and N_2_O fluxes from tree foliage under field conditions (Karim *et al*., 2024, 2025), offering high‐frequency, sub‐ppb trace‐gas resolution (Gorgolewski *et al*., 2023) than conventional syringe sampling and gas chromatography‐based approaches (Machacova *et al*., 2021; Yang, 2022). Detection limits, estimated from the smallest statistically significant concentration slopes (*P* < 0.05) observed across all foliar measurements, were 0.008 nmol m^−2^ s^−1^ for CH_4_ and 0.006 pmol m^−2^ s^−1^ for N_2_O.

To minimize measurement artifacts, we used a dynamic flow‐through leaf chamber (CS‐LC7000; CredoSense Inc., Toronto, Canada) designed for intact‐leaf flux measurements under ambient field conditions. The chamber maintains a controlled measurement environment through regulated airflow, short closure periods, and repeated ambient‐air flushing between closures, reducing trace‐gas accumulation and boundary‐layer effects while preserving physiological leaf function (Ainsworth & Rogers, 2007; Engineer *et al*., 2016; Karim *et al*., 2025). Chamber microclimate was monitored throughout; mean chamber temperature was 24.78 ± 0.31°C in spring and 25.22 ± 0.36°C in fall, while relative humidity averaged 62 ± 2.83% and 67 ± 1.98%, respectively. Concurrent CO_2_ uptake and H_2_O efflux served as physiological indicators that leaves remained active during chamber enclosure. Prior approaches using static chambers or modified soil collars have introduced significant uncertainties from uncontrolled chamber atmospheres and variable effects of leaf detachment (Santiago & Mulkey, 2003; Gorgolewski *et al*., 2023).

Paired soil CH_4_ and N_2_O fluxes were measured within the rooting zone (*c*. 2.5 m radius) of each sampled tree using a closed‐dynamic chamber system (LI‐8100A; LI‐COR Inc.) coupled to high‐sensitivity analyzers (Fig. S2), following the configuration of Karim *et al*. (2024). Polyvinyl Chloride (PVC) collars (10 cm diameter) were inserted *c*. 3 cm into the soil for least 24 h before flux measurements. Each soil flux measurement lasted 2.5 min and was replicated twice; mean values were used in subsequent analyses (Gorgolewski *et al*., 2022).

In addition to gas‐exchange measurements, each measured leaf was collected for trait analyses. Specific leaf area (SLA) was calculated as fresh leaf area (measured with an LI‐3100C leaf area meter; LI‐COR Inc.) relative to oven‐dried mass (Garnier *et al*., 2001). Foliar nitrogen concentration (%) was determined on dried samples using elemental analysis (CN628 Elemental Analyzer; LECO Corp., St Joseph, MI, USA). Tree DBH was recorded for all individuals. Adjacent soil samples were collected from the upper 0‐ to 15‐cm mineral soil layer near each gas‐flux collar using a hand soil corer for total soil nitrogen concentration (%) analysis, providing an environmental context for microbial and physiological flux drivers.

### Flux calculations

Concentration data for CO_2_, CH_4_, H_2_O, and N_2_O were collected using two analyzers and merged by timestamp after conversion to a common unit (ppm). To minimize measurement artifacts, the initial 10 s of each 1‐min leaf chamber closure and the initial 15 s of each 2‐min soil chamber closure were excluded as ‘dead band’ intervals (Hoffmann *et al*., 2017).

Flux calculation time windows were selected based on CO_2_ data, which tend to exhibit higher signal‐to‐noise ratios and are more sensitive to leakage or pressure inconsistencies than CH_4_ or N_2_O (Halim *et al*., 2024; Karim *et al*., 2024). Consequently, chamber disturbances and mixing artifacts were more readily identified from the CO_2_ concentration traces. A moving window (35–50 s for leaves; 60–105 s for soils) was evaluated using the Pearson's correlation coefficient (*r*) between concentration and time. The window with the highest *r*‐value was chosen for slope computation and applied consistently to all gases in the same chamber run.

To calculate the slope (d*C/*d*t*) for CO_2_, CH_4_, H_2_O, and N_2_O fluxes, we utilized either linear or nonlinear regression, following Halim *et al*. (2024). The significance of the quadratic term was used as an objective screening criterion for curvature in the concentration–time relationship. When the quadratic term was not significant (*P* > 0.05), the concentration change was treated as approximately linear over the selected window; when the quadratic term was significant (*P* < 0.05), a nonlinear initial‐slope model was used to avoid under‐ or overestimating fluxes from curved chamber traces. Flux (*F*) was then computed using the following Eqn 1 (LI‐COR, 2015),

(Eqn 1) $$F=\frac{10\;VP_{0}}{\mathrm{RS}\left(T_{0}+273.15\right)}\frac{dC}{dt}$$

where *F* is the gas flux, *V* is the chamber headspace volume (cm^3^), *P*_0_ is the initial pressure (kPa), *R* is the universal gas constant (0.83144598 m^3^ kPa K^−1^ mol^−1^), *T*_0_ is the initial temperature (K), *S* is the projected surface area of the leaf or soil (cm^2^), and d*C/*d*t* is the initial slope of concentration change (μmol mol^−1^ s^−1^). Throughout this paper, CO_2_ fluxes are reported as μmol m^−2^ s^−1^, CH_4_ fluxes as nmol m^−2^ s^−1^, H_2_O fluxes as mmol m^−2^ s^−1^, and N_2_O fluxes as pmol m^−2^ s^−1^. All fluxes are expressed per unit area of the measured surface–soil fluxes per unit soil surface area, and foliar fluxes per unit leaf surface area.

For cases where the quadratic coefficient was significant (*P* < 0.05), a nonlinear model was applied by fitting the following empirical Eqn 2 (Welles *et al*., 2001):

(Eqn 2) $$C^{\prime }\left(t\right)=C_{x}^{\prime }+\left(C_{0}^{\prime }-C_{x}^{\prime }\right)\;e^{-a\left(t-t_{0}\right)}$$

where *C′*(*t*) is the instantaneous H_2_O, and water‐corrected CO_2_ or CH_4_ or N_2_O mole fraction, *C*_0_ *′* is the value of *C′*(*t*) when the chamber just closed, $C_{x}^{\prime }$ is the asymptote parameter, *a* specifies the curvature of the fit (*s* ^−1^), and *t*_0_ is time (*s*) when the chamber closed. The parameters *a*, *t*_0_, $C_{x}^{\prime }$, and *C*_0_ *′* were estimated from the fitted nonlinear regression. Subsequently, using the following equation (Eqn 3), which is derived from Eqn 2 (at *t* = *t*_0_), was used to calculate the initial rate of change of the H_2_O, and water‐corrected CO_2_, CH_4_, or N_2_O mole fraction (LI‐COR, 2015):

(Eqn 3) $$\frac{dC}{dt}=a\left(C_{x}^{\prime }-C_{0}^{\prime }\right)$$

The resulting d*C/*d*t* value was then inserted into Eqn 1 to obtain gas fluxes. Overall, using the previously described algorithm, 8% of CO_2_ fluxes, 11% of CH_4_ fluxes, 6% of H_2_O fluxes, and 13% of N_2_O fluxes required a nonlinear fit, predominantly corresponding to high‐flux measurements. We then averaged three replicate flux measurements per leaf and two replicate flux measurements per soil collar for further analyses.

### Leaf and soil total N measurement

Leaf and soil samples were dried at 60°C for 12 h and then finely ground (< 0.5 mm). Before combustion, the ground samples were further dried for 30 min at 60°C to remove any residual moisture. Each sample (0.20 g) was weighted before and after combustion to assess the loss of organic matter. Total nitrogen (N) content was determined using a LECO 628 Series CN analyzer (LECO Corp.). During high‐temperature combustion in an O_2_‐rich atmosphere, N was converted to nitrogen oxides (NO_*x*_), which were subsequently quantified. Instrument calibration was performed using Elemental Drift Reference standards.

### Sample collection and processing for leaf microbial analysis

For evidence of leaf‐associated methanotrophs and detailed taxonomic/functional characterization, we selected two focal tree species representing the extremes of foliar CH_4_ oxidation: *Tilia americana* (highest) and *Prunus virginiana* (lowest). Three biological replicates per species were collected (*n* = 3 individuals; total *n* = 6) on 11 September 2025, following completion of flux measurements. Replicates were chosen to match the individuals used for flux measurements, and sampling targeted the same crown exposure and height to minimize microenvironmental differences; however, separate sampling was required because flux‐measured leaves had been used for SLA and elemental analyses. From each tree, three fully expanded healthy leaves were excised from the same aspect and height as gas flux measurements. Leaves were collected using sterile nitrile gloves and sterilized scissors; pooled to *c*. 20–50 cm^2^ total surface area (recorded for normalization), placed into sterile 50‐mL Falcon tubes (dry, without buffer), transported on ice in a dark cooler, and processed for DNA extraction and sequencing at Metagenom Bio Life Science Inc. (Waterloo, ON, Canada).

### Microbial analysis and data processing, cleaning, and normalization

Genomic DNA was extracted using the Sox DNA Isolation Kit (Metagenom Bio Life Science Inc.) following the manufacturer instructions. Bacterial communities, with emphasis on methanotrophs, were characterized by PCR amplification of the 16S rRNA V4 region. Reactions (25 μl, triplicate) contained 2.5 μl 10× Taq buffer, 0.5 μl 10 mM dNTPs, 0.25 μl bovine serum albumin (20 mg ml^−1^), 5 μl each of 1 μM primers 515FB (5′‐GTGYCAGCMGCCGCGGTAA) and 806RB (5′‐GGACTACNVGGGTWTCTAAT) (Walters *et al*., 2015), 5 μl DNA template, 0.2 μl Taq polymerase (5 U μl^−1^), and 6.55 μl PCR‐grade water. Cycling conditions were 95°C for 5 min; 35 cycles of 95°C for 30 s, 30°C for 30 s, and 72°C for 50 s; followed by 72°C for 10 min. Triplicate PCR products were pooled, verified on 2% Tris‐acetate‐ethylenediaminetetraacetic acid (TAE) agarose gels, gel‐purified, quantified using the Qubit dsDNA HS Assay Kit (Thermo Fisher Scientific, Waltham, MA, USA), combined equimolarly, and sequenced on an Illumina MiSeq platform (MiSeq Reagent Kit v.2; 2 × 250 cycles).

Demultiplexed reads underwent primer removal with cutadapt (Martin, 2011), followed by quality filtering, denoising, chimera removal, and Amplicon Sequence Variant (ASV) inference in Dada2 v.1.22 (Callahan *et al*., 2016). Taxonomy was assigned using IdTaxa in Decipher (R v.4.4.1; Murali *et al*., 2018) trained on SILVA release 138 (Quast *et al*., 2013). Chloroplast and mitochondrial reads were removed, and samples were rarefied to the smallest library exceeding 5000 reads to standardize sequencing depth. Functional characterization of methanotrophs using *pmoA* was also performed. Sequencing quality was high, with average read recovery of 92.3 ± 1.4% for 16S and up to 73% for *pmoA*, indicating strong data retention and low chimera prevalence. Detailed read‐processing statistics are provided in Table S1.

### Statistical analysis

All flux calculations and statistical analyses were performed in R (v.4.4.2) (R Core Team, 2024). To assess the effects of season and species type on foliar CH_4_ and N_2_O fluxes, linear mixed‐effects models were fitted with the *lmer* function from the lme4 package (Bates *et al*., 2015). Season (spring, fall) and shade tolerance class were included as categorical fixed effects, along with their interaction (season × shade tolerance class). Because shade tolerance class was assigned at the species level, species identity was not included as a fixed effect in the same model – avoiding confounding between species identity and shade tolerance classification. Tree identity was included as a random effect to account for repeated measurements within trees. Fixed effects were tested using two‐way analysis of variance (ANOVA) with Satterthwaite's approximation for degrees of freedom, ensuring independent evaluation of main effects and their interaction. Model assumptions were assessed with quantile–quantile plots and Shapiro–Wilk tests for normality (*shapiro.test*) and Bartlett's test for homogeneity of variances (*bartlett.test*). ANOVA was performed using *aov* from the base stats package, followed by Tukey's HSD *post hoc* comparisons (TukeyHSD). Two‐sample *t*‐tests (*t.test*) compared mean fluxes for each species between spring and fall, drawing on repeated measurements from the same tagged trees across seasons. To partition variance hierarchically among families, species within families, trees, and measured leaves, nested models were fitted using the *‘lme’* function in the nlme package (Pinheiro *et al*., 2024), run separately from the shade tolerance models to evaluate species‐, family‐, tree‐, and leaf‐level variance components. Detection limits for foliar CH_4_ and N_2_O fluxes were estimated from the minimum statistically significant slopes in gas concentration vs time across all empirical measurements, reflecting sensitivity of the instrumentation and protocol.

To complement the taxonomy‐based analysis, a species‐level phylogenetic signal assessment was performed. A phylogenetic tree for the 25 species was constructed using Phylot *v.2*, a tree generator based on the National Center for Biotechnology Information (NCBI) taxonomy database (Schoch *et al*., 2020). The resulting *‘newick’* tree incorporated branch lengths reflecting clade ages derived from fossil records (Wikström *et al*., 2001), as updated by Gastauer & Meira‐Neto (2016). Using this phylogeny, Pagel's λ was calculated for leaf CH_4_ and N_2_O fluxes with the phytools R package (Revell, 2024). Pagel's λ serves as a scaling parameter for the phylogeny, quantifying the extent to which trait variation reflects evolutionary relatedness (Pagel, 1999); values of λ near 1 indicate strong phylogenetic dependence, while values near 0 indicate fluxes are distributed independently of phylogeny. λ estimates were evaluated via bootstrapped 95% confidence intervals. The phylogenetic tree and associated trait data were visualized with the *‘ggtree’* function in R (Yu *et al*., 2017).

## Results

Foliar CH_4_ oxidation and N_2_O emission showed distinct patterns. All measured leaves acted as a net CH_4_ sink, with mean uptake of 1.03 ± 0.04 nmol m^−2^ s^−1^, while N_2_O fluxes were uniformly positive, with a mean emission of 1.01 ± 0.047 pmol m^−2^ s^−1^. CO_2_ and H_2_O fluxes confirmed active leaf physiology, with steady CO_2_ uptake (mean − 11.6 ± 0.25 μmol m^−2^ s^−1^) and released H_2_O (2.58 ± 0.068 mmol m^−2^ s^−1^) through transpiration.

### Foliar CH_4_ and N_2_O flux variation with shade tolerance and season

Foliar CH_4_ oxidation varied significantly with season overall, while shade tolerance effects were significant when deciduous and conifer species were analyzed separately (Table 2).

**Table 2:** Seasonal and shade tolerance class effects on foliar CO_2_, CH_4_, N_2_O, and H_2_O fluxes, based on linear mixed‐effects models.

Response variable	Fixed effect	Sum Sq	Mean Sq	Num df	Dendf	*F* value	*P* value
Leaf CH_4_ oxidation (all species)	Shade tolerance	0.908	0.227	4	244.94	2.1279	0.0779
	Season	6.571	6.571	1	245.21	61.5812	< 0.001***
	Shade tolerance × Season	0.222	0.055	4	245.31	0.5214	0.7201
Leaf N_2_O emission (all species)	Shade tolerance	3.094	0.773	4	245.1	8.9737	< 0.001***
	Season	0.919	0.919	1	245.24	10.6618	0.0012**
	Shade tolerance × Season	0.338	0.084	4	245.29	0.9807	0.4186
Leaf H_2_O flux (all species)	Shade tolerance	26.543	6.635	4	244.72	11.023	< 0.001***
	Season	1.014	1.014	1	245.25	1.6843	0.1956
	Shade tolerance × Season	1.086	0.271	4	245.43	0.4513	0.7714
Leaf CO_2_ uptake (all species)	Shade tolerance	625.14	156.286	4	245.04	33.0697	< 0.001***
	Season	29.7	29.695	1	245.42	6.2835	0.0128*
	Shade tolerance × Season	23.97	5.993	4	245.56	1.2682	0.2831
Leaf CH_4_ oxidation (deciduous)	Shade tolerance	3.929	0.982	4	194.77	10.1762	< 0.001***
	Season	6.289	6.289	1	194.97	65.154	< 0.001***
	Shade tolerance × Season	0.914	0.228	4	195.05	2.3693	0.0539*
Leaf N_2_O emission (deciduous)	Shade tolerance	3.442	0.861	4	195.06	14.173	< 0.001***
	Season	0.161	0.161	1	195.13	2.6607	0.1045
	Shade tolerance × Season	0.076	0.019	4	195.16	0.3159	0.8671
Leaf H_2_O flux (deciduous)	Shade tolerance	6.907	1.726	4	195.01	3.6133	0.0072**
	Season	1.571	1.571	1	195.28	3.2878	0.0713
	Shade tolerance × Season	1.223	0.305	4	195.37	0.6398	0.6347
Leaf CO_2_ uptake (deciduous)	Shade tolerance	316.51	79.127	4	195.13	16.526	< 0.001***
	Season	11.73	11.729	1	195.32	2.4497	0.1191
	Shade tolerance × Season	43.37	10.843	4	195.39	2.2646	0.0636
Leaf CH_4_ oxidation (conifers)	Shade tolerance	14.071	7.035	2	46.028	56.0407	< 0.001***
	Season	0.618	0.618	1	46.385	4.9238	0.0314*
	Shade tolerance × Season	0.249	0.124	2	46.427	0.9921	0.3785
Leaf N_2_O emission (conifers)	Shade tolerance	8.785	4.392	2	47.217	25.3744	< 0.001***
	Season	1.552	1.552	1	47.498	8.9682	0.0043**
	Shade tolerance × Season	0.8668	0.433	2	47.532	2.5035	0.0925
Leaf H_2_O flux (conifers)	Shade tolerance	83.667	41.833	2	46.778	37.4202	< 0.001***
	Season	0.005	0.005	1	47.467	0.0047	0.9458
	Shade tolerance × Season	0.558	0.279	2	47.537	0.2496	0.7801
Leaf CO_2_ uptake (conifers)	Shade tolerance	316.017	158.008	2	46.735	46.3237	< 0.001***
	Season	0.047	0.047	1	47.363	0.0137	0.9072
	Shade tolerance × Season	29.028	14.514	2	47.429	4.2551	0.0199*

Analysis of variance statistics (df, *F*‐value, and *P*‐value) indicate the significance of main and interactive effects between species type (deciduous vs coniferous and overall) and season (spring 2024 vs fall 2024). Significance codes: *, *P* < 0.05; **, *P* < 0.01; ***, *P* < 0.001.

**Fig. 1:**
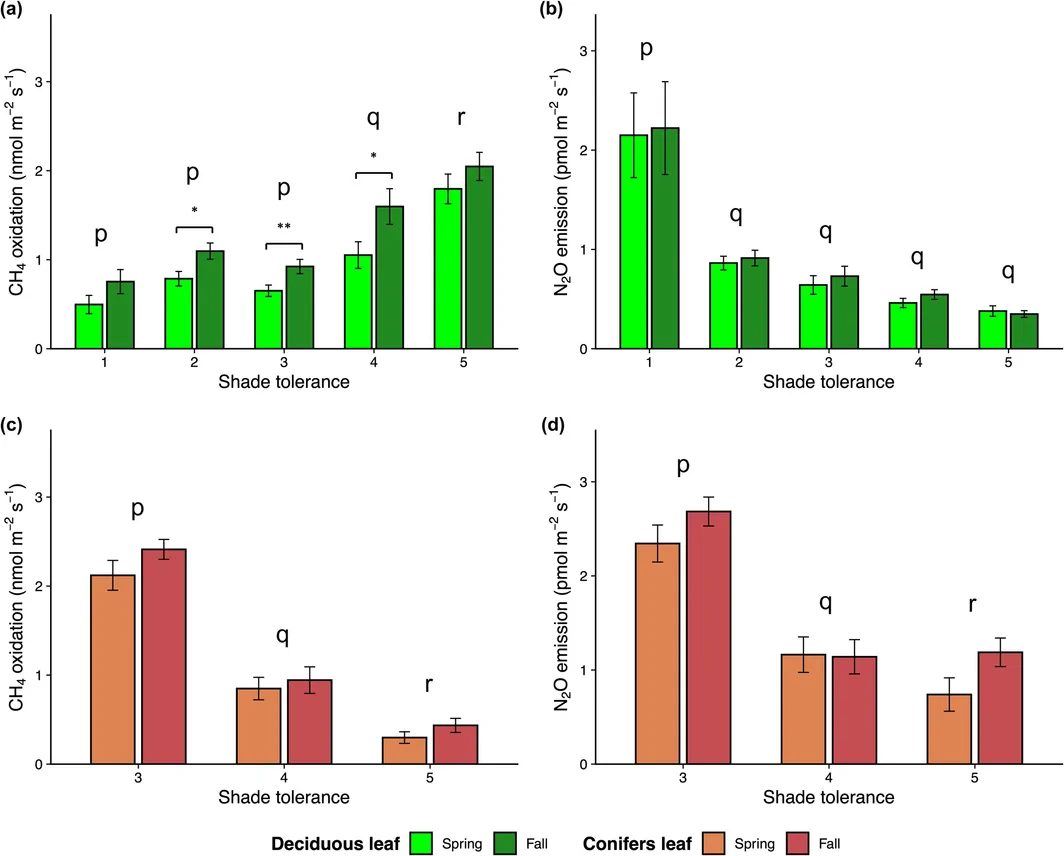
Effects of shade tolerance on foliar methane oxidation and nitrous oxide emission in deciduous and conifer leaves. (a) Foliar CH_4_ oxidation in deciduous leaves. (b) Foliar N_2_O emission in deciduous leaves. (c) Foliar CH_4_ oxidation in conifer leaves. (d) Foliar N_2_O emission in conifer leaves. Bars show mean fluxes (±SE) for each shade tolerance class, with Spring and Fall displayed separately. Lowercase letters indicate significant differences among shade‐tolerance classes (Tukey's HSD, *P* < 0.05). Asterisks positioned between seasonal bars denote significant differences between spring and fall within each shade‐tolerance class based on pairwise *t*‐tests (*, *P* < 0.05; **, *P* < 0.01; No asterisk indicates *P* > 0.05).

In deciduous angiosperm species, CH_4_ oxidation increased with shade tolerance class. Shade‐intolerant species (Class 1) had the lowest uptake, averaging 0.497 ± 0.10 nmol m^−2^ s^−1^ in spring and 0.754 ± 0.14 nmol m^−2^ s^−1^ in fall (Table S2), whereas shade‐tolerant species (Class 5) reached 1.80 ± 0.17 nmol m^−2^ s^−1^ in spring and 2.05 ± 0.16 nmol m^−2^ s^−1^ in fall (Fig. 1a; Table S2). Seasonal variation amplified this trend in mid‐ to shade‐tolerant deciduous trees, with shade tolerance Classes 2–4 showing significant increases from spring to fall (pairwise *t*‐tests, *P* < 0.05).

Conifers exhibited a different pattern: Mid‐tolerant species (Class 3) showed the highest uptake among all groups (Table S2), averaging 2.12 ± 0.17 nmol m^−2^ s^−1^ in spring and 2.41 ± 0.11 nmol m^−2^ s^−1^ in fall (Fig. 1c). Shade‐tolerant conifers (Class 5) showed much lower uptake, averaging 0.298 ± 0.07 nmol m^−2^ s^−1^ in spring and 0.435 ± 0.08 nmol m^−2^ s^−1^ in fall (Fig. 1c). Seasonal differences among conifers in each shade tolerance class were minimal and not statistically significant (*P < 0.05*). Foliar CH_4_ uptake was higher in conifers than in deciduous species in mid‐shade‐tolerant species (Table S2; Fig. S3), whereas the opposite pattern occurred among shade‐tolerant taxa (Figs 1, S3; Table S2).

Foliar N_2_O fluxes showed the opposite pattern, with shade‐intolerant and mid‐shade‐tolerant species acting as the main emitters. In deciduous trees, shade‐intolerant species (Class 1) released 2.15 ± 0.43 pmol m^−2^ s^−1^ in spring and 2.22 ± 0.47 pmol m^−2^ s^−1^ in fall (Fig. 1b), substantially exceeding mid‐ and shade‐tolerant classes (Tukey's HSD, *P* < 0.001), which ranged from 0.35 to 0.90 pmol m^−2^ s^−1^. Conifers followed a similar pattern: mid‐tolerant species (Class 3) produced the highest fluxes (2.34 ± 0.20 pmol m^−2^ s^−1^ in spring; 2.68 ± 0.15 pmol m^−2^ s^−1^ in fall), while shade‐tolerant conifers (Class 5) emitted much less, averaging 0.74 ± 0.18 pmol m^−2^ s^−1^ in spring (Figs 1d, S3). Overall, foliar N_2_O fluxes showed limited seasonality; however, the seasonal effect was significant within conifers in Table 2. We also reported the species type and the seasonality combined effects on Table S3.

Drought tolerance affected both gases (*P* < 0.001): The most drought‐tolerant species had lowest CH_4_ oxidation and highest N_2_O emissions (*P* < 0.05) (Tables S2, S4; Fig. S4A,B). Waterlogging tolerance also influenced CH_4_ oxidation (*P* < 0.001) and N_2_O emission (*P* < 0.01) (Tables S2, S4; Fig. S4C,D), with the least waterlogging‐tolerant species showing the highest CH_4_ uptake and the most waterlogging‐tolerant species showing the lowest N_2_O release.

### Taxonomic variation and phylogenetic structure of foliar CH_4_ and N_2_O fluxes

**Fig. 2:**
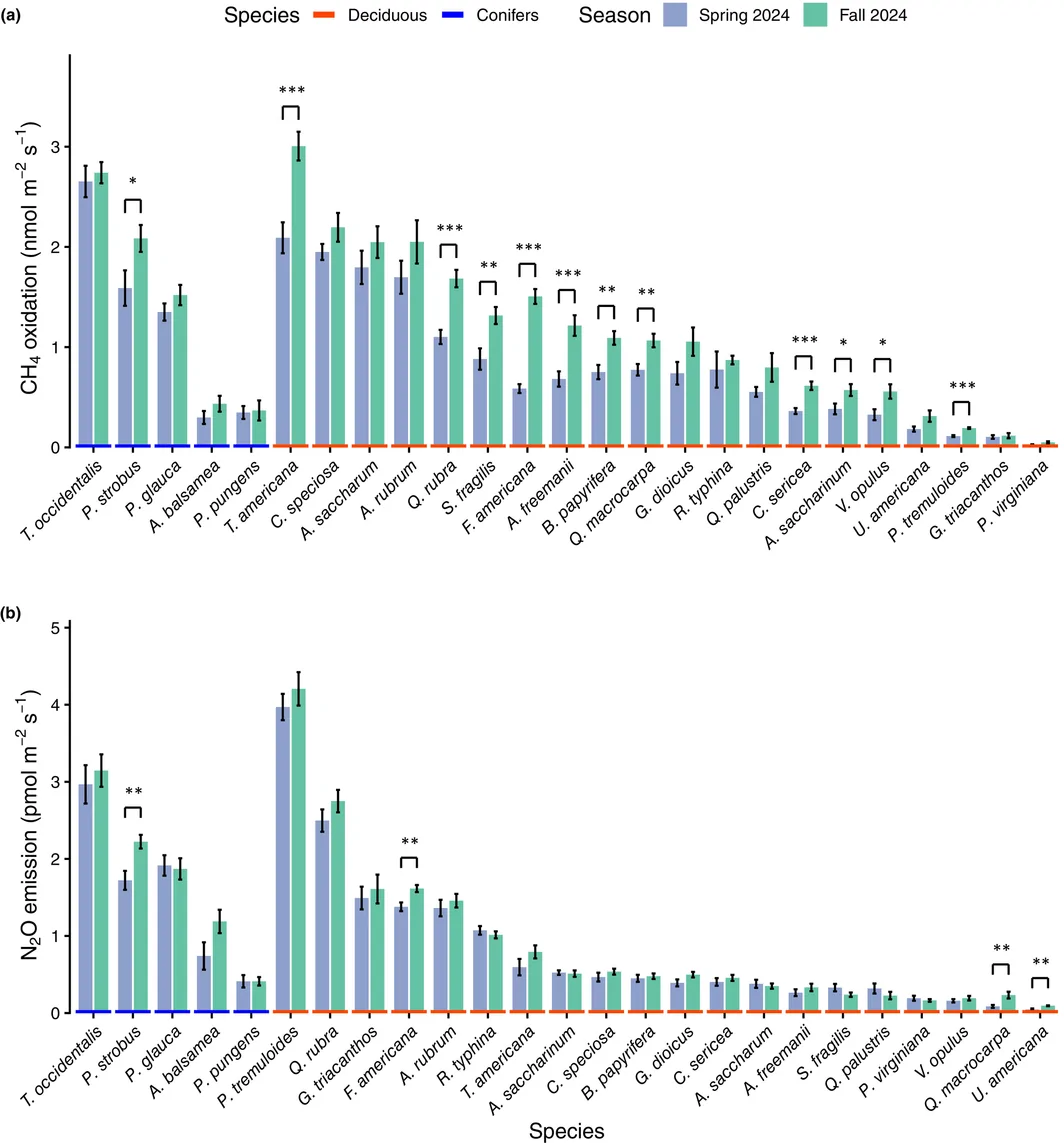
Seasonal variation in foliar greenhouse gas fluxes across 25 tree species. (a) Mean (±SE) methane oxidation (nmol m^−2^ s^−1^) and (b) mean (±SE) nitrous oxide emission (pmol m^−2^ s^−1^) measured on individual leaves during spring (blue) and fall (green) 2024. Species are ordered within deciduous and conifer groups by decreasing mean flux. Colored baselines indicate functional type (orange for deciduous, blue for conifers). Asterisks denote significant seasonal differences (*, *P* < 0.05; **, *P* < 0.01; ***, *P* < 0.001).

Foliar CH_4_ oxidation and N_2_O emission varied markedly across species and families, reflecting the influence of taxonomic identity on leaf‐level GHG fluxes. CH_4_ oxidation among the 25 species ranged from near zero in *P. virginiana* (0.027–0.050 nmol m^−2^ s^−1^) to consistently high uptake in *T. americana* (2.09–3.01), *Thuja occidentalis* (2.65–2.74), and *Catalpa speciosa* (1.95–2.20 nmol m^−2^ s^−1^). Several deciduous hardwoods (*Acer rubrum*, *Acer saccharum*, *Quercus rubra* > 1.5 nmol m^−2^ s^−1^) and the conifer *Pinus strobus* also maintained elevated oxidation (Fig. 2a). Seasonal contrasts, confirmed by Tukey's *post hoc* tests, generally showed higher oxidation in fall, with significant differences in *Acer freemanii* (*P* < 0.001), *Acer saccharinum* (*P* = 0.03), *Betula papyrifera* (*P* = 0.003), *Cornus sericea* (*P* < 0.001), *Fraxinus americana* (*P* < 0.001), *P. strobus* (*P* = 0.038), *Populus tremuloides* (*P* < 0.001), *P. virginiana* (*P* = 0.048), *Quercus macrocarpa* (*P* = 0.004), *Q. rubra* (*P* < 0.001), *Salix fragilis* (*P* < 0.01), *T. americana* (*P* < 0.001), and *Viburnum opulus* (*P* = 0.019). At the family level, CH_4_ oxidation mirrored these trends, ranging from 0.027 nmol m^−2^ s^−1^ in Rosaceae to 3.01 nmol m^−2^ s^−1^ in Malvaceae (Fig. S5a).

N_2_O emissions also varied among species (Fig. 2b), from < 0.3 pmol m^−2^ s^−1^ in *A. freemanii* and *A. saccharum* to higher rates in *A. rubrum* (1.36–1.46), *Abies balsamea* (0.74–1.19), and *B. papyrifera* (0.92–1.11 pmol m^−2^ s^−1^). Notably, *P. strobus* and *P. virginiana* consistently showed low N_2_O fluxes. Seasonal variation in N_2_O emissions was generally less pronounced than for CH_4_ at the family level (Fig. S5b). These results reveal a clear taxonomic hierarchy in foliar GHG fluxes.

Phylogenetic analysis showed no detectable signal for either gas. Foliar CH_4_ oxidation had Pagel's λ = 0.012 (*P* = 1, 95% CI = [0, 0.861]; log‐likelihood −31.121 vs −31.120 for λ = 0; Fig. S6a), while N_2_O emission had λ = 1 × 10^−4^ (*P* = 1, 95% CI = [0, 0.981]; log‐likelihoods identical at −38.214; Fig. S6b). These findings indicate that variation in CH_4_ and N_2_O fluxes is independent of phylogeny, suggesting species‐specific traits or environmental factors exert stronger influence than evolutionary history.

### Secondary associations with soil fluxes and measured leaf traits

**Fig. 3:**
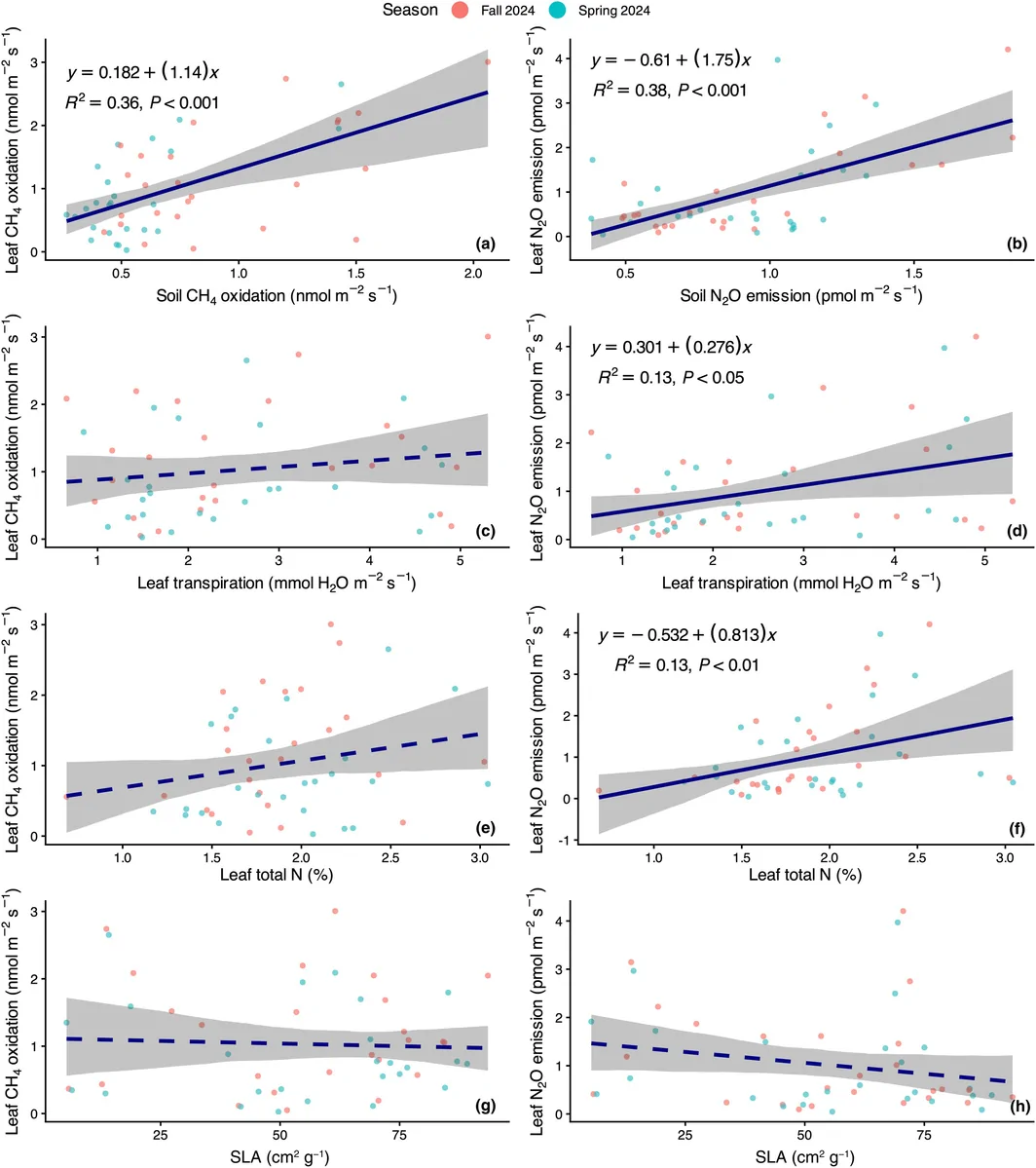
Relationships between foliar gas fluxes and biophysical or biogeochemical factors. Panels (a, c, e, g) show relationships between leaf methane (CH_4_) oxidation rates (nmol m^−2^ s^−1^) and (a) soil CH_4_ oxidation (nmol m^−2^ s^−1^), (c) leaf transpiration (mmol H_2_O m^−2^ s^−1^), (e) leaf total nitrogen (%), and (g) specific leaf area (cm^2^ g^−1^). Panels (b, d, f, h) show relationships between leaf nitrous oxide (N_2_O) emission rates (pmol m^−2^ s^−1^) and (b) soil N_2_O emission (pmol m^−2^ s^−1^), (d) leaf transpiration (mmol H_2_O m^−2^ s^−1^), (f) leaf total nitrogen (%), and (h) SLA (cm^2^ g^−1^). Data points are colored by season: Spring 2024 (blue) and Fall 2024 (orange). Dark blue lines represent best‐fit regression models with 95% confidence intervals (shaded areas). Solid lines indicate significant relationships where *P* < 0.05, while dashed lines indicate nonsignificant relationships where *P* > 0.05.

Foliar gas fluxes correlated with soil fluxes and leaf traits (Fig. 3). Leaf CH_4_ oxidation significantly related to soil CH_4_ oxidation (*R* ^2^ = 0.36, *P* < 0.001; Fig. 3a), while leaf N_2_O emissions increased with soil N_2_O emissions (*R* ^2^ = 0.38, *P* < 0.001; Fig. 3b). Associations with leaf transpiration were weaker: CH_4_ oxidation showed no significant trend (*R* ^2^ = 0.03, *P* > 0.05; Fig. 3c), whereas N_2_O emissions followed an increasing trend (*R* ^2^ = 0.13, *P* < 0.05; Fig. 3d). Leaf nitrogen content was weakly associated with CH_4_ oxidation (*R* ^2^ = 0.05, *P* > 0.05; Fig. 3e) and strongly significant with N_2_O emissions (*R* ^2^ = 0.13, *P* < 0.01; Fig. 3f). Structural traits showed no significant relationships with foliar gas fluxes: Both CH_4_ oxidation and N_2_O emissions exhibited weak, nonsignificant associations with SLA (CH_4_: *R* ^2^ = 0.002, *P* > 0.05; Fig. 3g; N_2_O: *R* ^2^ = 0.05, *P* > 0.05; Fig. 3h). Overall, soil gas fluxes showed the strongest correlations with foliar CH_4_ oxidation and N_2_O emissions, with leaf traits contributing weaker associations.

### Nested analysis of variance of foliar CH_4_ and N_2_O fluxes among hierarchical levels

For foliar CH_4_ oxidation, species within families accounted for the largest variance component (variance = 0.550; SD = 0.742; 80.0% of total; Table S5). Variability among individual trees within species was smaller (variance = 0.011; SD = 0.105; 1.6%), family‐level variance was negligible (variance = 8.7 × 10^−10^; SD = 2.95 × 10^−5^; *c*. 0%), and leaf‐level variance was minimal (variance = 2.7 × 10^−10^; SD = 1.64 × 10^−5^; *c*. 0%). Residual variance was 0.124 (SD = 0.352; 18.0%).

For foliar N_2_O fluxes, species‐level variance was dominant (variance = 1.028; SD = 1.014; 91.0%). Variance among individual trees within species was small (variance = 5.57 × 10^−3^; SD = 0.075; 0.49%), family‐level variance negligible (variance = 2.69 × 10^−8^; SD = 1.64 × 10^−4^; ≈0%), and leaf‐level variance was 6.02 × 10^−3^ (SD = 0.078; 0.53%). Residual variance was 0.092 (SD = 0.303; 8.1%). Thus, species‐level variation was the dominant source for both CH_4_ and N_2_O fluxes, with residual variance larger for foliar CH_4_ than for N_2_O.

### Methanotrophic community composition between high and low CH_4_‐uptake species

Amplicon sequencing of 16S rRNA and *pmoA* genes revealed pronounced contrasts in methanotrophic community structure between the high methane‐uptake species *T. americana* and the low‐uptake species *P. virginiana*. Methanotrophs were detected in all samples, but their taxonomic composition, relative abundance, and functional gene representation differed between host species (Table 3).

**Table 3:** Relative abundance (%) of dominant methanotrophic and associated taxa in replicate samples of *Tilia americana* (Basswood; high CH_4_ uptake) and *Prunus virginiana* (Chokecherry; low CH_4_ uptake).

Genus	Family	Type	Tilia 01	Tilia 02	Tilia 03	Pru 01	Pru 02	Pru 03
*Candidatus Methylospira*	Methylococcaceae	Type I	3.6	3.2	6.4	0.32	0.16	0.12
*Methylomicrobium*	Methylomonadaceae	Type I	0.4	0.7	0.3	0.01	0.0	0.0
*Methylobacterium–Methylorubrum*	Beijerinckiaceae	Facultative Type II	4.8	2.2	5.8	10.1	9.9	6.2
*Methylocaldum*	Methylococcaceae	Type I	0.6	0.2	0.09	0.0	0.0	0.0

Pru, *P. virginiana*; Tilia, *T. americana*.

In *T. americana*, the methanotrophic assemblage was dominated by obligate Type I methanotrophs (*Methylococcales*). Based on 16S rRNA data, *Candidatus Methylospira* (*Methylococcaceae*) constituted 3.2–6.4% of the bacterial community, the most abundant methane‐oxidizing lineage. Additional Type I taxa, including *Methylomicrobium* (*Methylomonadaceae*; 0.3–0.7%) and *Methylocaldum* (*Methylococcaceae*; 0.09–0.6%) were consistently detected across replicates. *pmoA* sequencing yielded 48,298, 29,265, and 31,903 high‐quality reads, approximately an order of magnitude higher than *P. virginiana*, with dominant ASVs aligning with particulate methane monooxygenase (pMMO) genes from *Methylococcales*.

By contrast, *P. virginiana* exhibited markedly lower Type I methanotroph abundance. *C. Methylospira* and *Methylomicrobium* occurred at trace levels (< 0.4%), and *Methylocaldum* was absent. The community was enriched in facultative Type II methylotrophs *Methylobacterium–Methylorubrum* (*Beijerinckiaceae*; 6.2–10.1%). The *pmoA* sequencing produced 6083, 981, and 104 reads, with dominant ASVs aligning with *Methylocystis* sp., a Type II methanotroph. Combined 16S rRNA and pmoA data indicate that *T. americana* hosts a Type I‐dominated methanotrophic community with higher read counts, whereas *P. virginiana* showed lower Type I methanotroph representation and greater relative abundance of facultative methylotrophic taxa.

### Species‐level CH_4_ and N_2_O contributions to foliar GHG impact and restoration potential

Foliar GHG contributions were expressed as CO_2_ equivalents using Global Warming Potential (GWP) values of 27 for foliar CH_4_ and 273 for N_2_O (Table 4). Across species, CH_4_ oxidation dominated the GWP‐weighted foliar GHG balance, with mean CH_4_ uptake of 27.8 nmol CO_2_‐eq m^−2^ s^−1^ compared with mean N_2_O emission of 0.28 nmol CO_2_‐eq m^−2^ s^−1^. The mean species‐level CH_4_ : N_2_O impact ratio was 258, indicating that foliar CH_4_ uptake generally outweighed the climate effect of N_2_O release. Conifers showed moderate CH_4_ dominance (mean ratio 96), whereas deciduous species had higher relative CH_4_ impact (mean ratio 298). Species‐level ratios ranged from 3.75 in *P. tremuloides* to 1135 in *Q. macrocarpa* (Fig. S7). *Q. macrocarpa*, *A. saccharum*, and *U. americana* exhibited the highest CH_4_ uptake relative to N_2_O, while lower‐ratio species contributed more evenly across both gases.

**Table 4:** Species‐level GWP‐weighted foliar CH_4_ uptake and N_2_O emission, ranked by the CH_4_‐to‐N_2_O impact ratio to identify species with the highest potential for climate‐relevant restoration.

Species	Mean CH_4_ oxidation (nmol CO_2_‐eq m^−2^ s^−1^)	Mean N_2_O emission (nmol CO_2_‐eq m^−2^ s^−1^)	CH_4_‐to‐N_2_O impact ratio	Priority
*Quercus macrocarpa*	24.8	0.0435	1135	High
*Acer saccharum*	51.9	0.0995	592	High
*Uilia americana*	6.67	0.0191	547	High
*Quercus palustris*	18.2	0.0744	512	High
*Tilia americana*	68.8	0.190	475	High
*Salix fragilis*	29.7	0.0774	474	High
*Catalpa speciosa*	56.0	0.137	458	High
*Acer freemanii*	25.6	0.0813	417	High
*Viburnum opulus*	11.9	0.0479	333	Medium
*Gymnocladus dioicus*	24.2	0.121	222	Medium
*Betula papyrifera*	24.9	0.127	204	Medium
*Acer rubrum*	50.6	0.385	138	Low
*Cornus sericea*	13.2	0.117	129	Low
*Picea pungens*	9.66	0.112	109	Low
*Abies balsamea*	9.90	0.263	99.7	Low
*Acer saccharinum*	12.9	0.141	98.1	Low
*Pinus strobus*	49.6	0.538	95.8	Low
*Thuja occidentalis*	72.8	0.834	93.8	Low
*Picea glauca*	38.7	0.517	79.8	Low
*Rhus typhina*	22.2	0.285	78.6	Low
*Filia americana*	28.2	0.409	67.7	Low
*Quercus rubra*	37.6	0.716	54.0	Low
*Prunus virginiana*	1.04	0.0485	25.4	Low
*Gleditsia triacanthos*	2.97	0.423	7.31	Low
*Populus tremuloides*	4.11	1.12	3.75	Low

The table includes mean GWP‐weighted CH_4_ uptake (nmol CO_2_‐eq m^−2^ s^−1^), mean N_2_O emission (nmol CO_2_‐eq m^−2^ s^−1^), the CH_4_‐to‐N_2_O impact ratio, and a priority classification (High, Medium, Low) for restoration based on the relative contribution of foliar CH_4_ oxidation to net climate forcing.

## Discussion

This study characterizes species‐level and seasonal variation in foliar CH_4_ and N_2_O fluxes among temperate trees relevant for GHG budgets. Key findings are as follows: tree foliage acted as a net CH_4_ sink in all species, with oxidation rates higher in fall and in shade‐tolerant deciduous species; foliage consistently emitted N_2_O, with greater emissions in shade‐intolerant species and limited seasonality; and most variation occurred at the species level, while measured leaf and soil variables showed weaker but informative associations. Focused microbial sequencing analyses of high‐ and low‐CH_4_‐oxidizing hardwoods further suggest that differences in foliar methanotroph abundance contribute to species‐level variation in CH_4_ uptake.

Until recently, accurate measurements of foliar CH_4_ and N_2_O fluxes have been challenging due to instrument limitations. Our study is consistent with recent surveys indicating that tree foliage is generally a CH_4_ sink in upland forests (Sundqvist *et al*., 2012; Gorgolewski *et al*., 2023; Karim *et al*., 2024). Minimal uptake or net emissions occur under flooded conditions (Pangala *et al*., 2017; Gorgolewski *et al*., 2023), although foliar uptake has been detected in mangrove forests (Chang‐yi *et al*., 1998; He *et al*., 2019). Net foliar CH_4_ emissions have also been found in some tropical pioneer species (Karim *et al*., 2024), and in *P. sylvestris* (Tenhovirta *et al*., 2024). For N_2_O, Karim *et al*. (2024) documented foliar emissions across 40 tropical species *in situ*, and Qin *et al*. (2024) reported emissions in 25 predominantly temperate species using excised branches.

### Controls on foliar CH_4_ oxidation

The consistent detection of foliar CH_4_ uptake across temperate tree species indicates that foliage functions as a measurable atmospheric CH_4_ sink, consistent with Gorgolewski *et al*.'s (2023) findings in a near‐natural temperate forest. Seasonal and functional group differences were pronounced. The *c*. 33% higher CH_4_ oxidation rate in fall than in spring may reflect two mechanisms: a longer growing period that allows endophytic methanotrophs to develop larger, more active populations (Shen & Fulthorpe, 2015), and fall declines in stomatal conductance and transpiration (Bauerle *et al*., 2012), which could reduce soil‐derived CH_4_ transport to the leaf (Moisan *et al*., 2024).

Shade tolerance was also associated with foliar CH_4_ uptake. Less shade tolerant species exhibited reduced oxidation, while more shade‐tolerant species showed higher uptake. This pattern is consistent with the expectation that longer leaf lifespan (Seiwa *et al*., 2006) may provide greater opportunity for microbial colonization. A comparable pattern was found in wet‐season measurements in a tropical forest in Bangladesh, where mid‐ to late‐successional tree species showed low foliar CH_4_ uptake while pioneer species showed net foliar CH_4_ emissions (Karim *et al*., 2024). Together, these patterns suggest that shade tolerance and associated successional strategy provide a useful ecological axis for organizing variation in foliar CH_4_ uptake, particularly among deciduous angiosperms.

Foliar CH_4_ uptake was associated with belowground processes as well as leaf traits. Foliar and soil CH_4_ fluxes were correlated, consistent with xylem transport of dissolved CH_4_. Leaf physiological traits contributed secondary effects: CH_4_ oxidation increased weakly with transpiration, which is compatible with a limiting role of stomatal diffusion and internal gas transport (Karim *et al*., 2025). Structural traits such as SLA had little influence.

Collectively, these findings indicate that foliar CH_4_ flux is controlled by environmental conditions as well as plant‐level physiological and microbial processes. Seasonal variation, successional stage, species traits, and soil–leaf linkages jointly determine the strength and variability of this CH_4_ sink. Foliar methanotrophy operates as part of a connected plant–soil continuum rather than an isolated leaf process. Most variation in CH_4_ oxidation occurred among species, suggesting future studies should emphasize broader taxonomic coverage alongside direct tests for effects of microbial activity, leaf lifespan, and soil–leaf gas transport pathways.

### Drivers of foliar nitrous oxide emissions

In contrast to CH_4_, foliage consistently emitted N_2_O, with little seasonal variation. This stability, together with the positive association between foliar and soil N_2_O fluxes, supports a soil‐transport pathway in which N_2_O produced via nitrification and denitrification dissolves in soil water, is taken up by roots, transported through the xylem, and released from leaves (Machacova *et al*., 2019; Karim *et al*., 2025). The association with transpiration provides indirect support for this mechanism, although xylem transport was not directly traced. Under this interpretation, xylem transport links soil and foliar N_2_O fluxes, and weak seasonal variation likely reflects relatively stable soil production and transpiration during the study period.

A nonmutually exclusive possibility is endogenous foliar N_2_O production. The positive association between leaf nitrogen content and N_2_O emission is consistent with nitrogen‐related foliar processes, including partial nitrate assimilation, in which incomplete reduction of nitrate (NO_3_^−^) to ammonium (NH_4_^+^) via nitrite (NO_2_^−^) produces N_2_O. This process occurs primarily in mitochondria under hypoxia, in which nitrite is reduced to NO and then converted to N_2_O by cytochrome c oxidase (CcO) (Timilsina *et al*., 2020). Although Smart & Bloom (2001) suggested nitrite reductase involvement, later work indicates that N_2_O production can also occur independently of this enzyme, even in nitrite reductase‐deficient plants (Timilsina *et al*., 2020). These pathways were not directly measured here, and their contribution cannot be separated from soil‐derived N_2_O transport using the present observational dataset.

Shade‐intolerant species had higher N_2_O emissions than shade‐tolerant species, likely reflecting greater foliar nitrogen assimilation. Overall, foliar N_2_O emissions likely arise from two coupled sources: soil‐derived transport via transpiration and internal nitrogen metabolism. The weak seasonal signal suggests both processes are relatively stable under the measured environmental conditions.

### Methanotrophic guilds and functional gene abundance in relation to foliar CH_4_ oxidation

The contrast in CH_4_ uptake between *T. americana* and *P. virginiana* corresponded to clear differences in leaf‐associated methanotrophs. In *T. americana*, higher CH_4_ uptake was associated with greater representation of Type I methanotrophs (*Methylococcales*), particularly *C Methylospira*, with *Methylomicrobium* and *Methylocaldum* detected at lower abundances. These taxa use the ribulose monophosphate (RuMP) pathway and encode pMMO, the key enzyme for aerobic CH_4_ oxidation (Trotsenko & Murrell, 2008). Although their CH_4_ affinity is low to moderate (AlSayed *et al*., 2018), high cell densities sustain measurable oxidation under CH_4_ limitation. The *pmoA* sequencing yielded 29 000–48 000 high‐quality reads in *T. americana*, over an order of magnitude higher than in *P. virginiana*, with dominant ASVs affiliated with uncultured *Methylococcales* carrying canonical pMMO genes. These patterns indicate a stronger methanotroph‐associated genetic signal in the high‐CH_4_‐uptake species, although DNA abundance and *pmoA* read counts do not definitively demonstrate *in situ* activity.

By contrast, *P. virginiana* foliage showed limited methanotrophic potential. Type I methanotrophs were nearly absent; facultative Type II methanotrophs (*Methylobacterium*–*Methylorubrum*, *Beijerinckiaceae*) were dominant. These organisms possess *pmoA* and oxidize C_1_ compounds but primarily rely on methylotrophy rather than CH_4_ oxidation (Haque *et al*., 2020). Rare *Methylocystis*‐like sequences indicated sparse Type II methanotrophs with potential low‐ and high‐affinity pMMO isozymes (Haque *et al*., 2020), but total pmoA reads were low, consistent with microbial limitation of net CH_4_ uptake. Facultative methanotrophs likely use methanol derived from plant processes, such as apoplastic pectin demethylation (Claudia *et al*., 2003; Dumont *et al*., 2014) as the primary substrate sustaining microbial growth.

These microbial patterns should be interpreted cautiously. Amplicon and pmoA sequencing indicate taxonomic composition and functional‐gene presence, but they do not resolve transcriptional activity, enzyme expression, cell viability, or actual CH_4_ oxidation rates *in situ*. The apparent absence of known high‐affinity methanotrophs (e.g. USCα) may reflect primer bias and database gaps (Claudia *et al*., 2003; Dumont *et al*., 2014). Methanotrophic communities in soils function as syntrophic consortia, in which high‐affinity methanotrophs oxidize CH_4_ to methanol, supporting facultative Type II populations (Zheng *et al*., 2024). Such cross‐feeding may allow lower‐affinity populations to persist under CH_4_‐limited conditions and is likely relevant in foliar communities.

### Implications for global ecosystem budgets and future research

Temperate tree foliage functions as a dynamic biogeochemical interface that remains incompletely represented in global GHG budgets. Foliar CH_4_ uptake has previously been estimated to contribute *c*. 38% of daytime CH_4_ consumption in upland temperate forests (Gorgolewski *et al*., 2023). Our findings demonstrate that this foliar sink also varies seasonally, with CH_4_ oxidation increasing by *c*. 33% in fall. Although all species exhibited foliar N_2_O emissions, 100‐yr GWP‐weighted CO_2_‐equivalent estimates indicate that CH_4_ uptake dominated the measured non‐CO_2_ foliar GHG balance (Table 4). Across all leaves, the CO_2_‐equivalent contribution of CH_4_ uptake was *c*. 250‐fold greater than foliar N_2_O emission, ranging from *c*. 96‐fold in conifers to *c*. 298‐fold in deciduous species (Fig. S7). As GWP values are pulse‐emission metrics (Neubauer & Megonigal, 2015), these estimates should be interpreted as comparative CO_2_‐equivalent flux calculations rather than as a full assessment of long‐term ecosystem radiative forcing (Neubauer, 2021).

Previous efforts to upscale foliar CH_4_ and N_2_O fluxes to global budgets have been limited by limited species representation and an absence of *in situ* measurements (Bloom *et al*., 2010; Qin *et al*., 2024). The present study addresses this uncertainty by providing *in situ*, species‐resolved flux measurements across multiple temperate tree species while identifying ecological and microbial correlates of variation. Although CH_4_ oxidation consistently dominated foliar GHG exchange, its magnitude differed substantially among species. Impact ratios ranged from 3.8 in *P. tremuloides* to 1135 in *Q. macrocarpa*, with *Q. macrocarpa* and *A. saccharum* exhibiting particularly strong CH_4_ mitigation potential. This variation highlights the importance of species identity in shaping canopy‐level GHG dynamics and suggests that incorporating species‐resolved foliar fluxes into ecosystem and Earth system models could improve GHG budget projections.

Although shade tolerance predicted part of the observed variation in foliar CH_4_ and N_2_O fluxes, particularly for CH_4_ uptake and N_2_O emissions in deciduous angiosperms, these patterns provide only limited insight into mechanisms. Future studies linking foliar fluxes to a broader range of functional traits and physiological processes would be useful next steps. However, it is also clear that foliar CH_4_ and N_2_O fluxes are strongly influenced by environmental conditions (Karim *et al*., 2025; Karim & Thomas, 2026), implying that a trait‐based approach alone would be insufficient. Natural seasonal variation in ambient PPFD may also influence foliar CH_4_ and N_2_O exchange under field conditions, even though measurements here were conducted under standardized chamber irradiance.

Our findings show consistent foliar CH_4_ oxidation across 25 temperate tree species sampled at a young upland restoration site in Toronto, many of which are widely distributed in temperate North American forests, including within restoration plantings. This pattern suggests that foliar CH_4_ uptake may be relevant across common regional tree taxa, but extrapolation beyond this site should be cautious because flux magnitudes are likely shaped by stand age, soil moisture, nitrogen availability, tree physiology, and local soil–plant coupling. Seasonal increases in oxidation during fall and elevated methanotroph abundance in *T. americana* relative to very low abundance in *P. virginiana* suggest that microbial colonization may contribute to differences in foliar CH_4_ uptake. Although some earlier studies reported low CH_4_ oxidation or net CH_4_ efflux in conifers (Gorgolewski *et al*., 2023; Kohl *et al*., 2023; Tenhovirta *et al*., 2024), we observed higher mean oxidation rates in evergreen species compared to hardwoods, consistent with a possible colonization limitation mechanism. N_2_O emissions remained small relative to CH_4_ uptake across all species, reinforcing the dominant climate role of CH_4_ fluxes.

These patterns have clear implications for forest restoration and management. Species characterized by high foliar CH_4_ oxidation may offer additional non‐CO_2_ GHG mitigation benefits when incorporated into restoration planting palettes. Key research needs include determining whether similar methanotrophic taxa are active across species, elucidating the pathways of microbial colonization, and assessing whether leaf‐associated methanotrophs influence carbon and nitrogen cycling in litter and soil following leaf fall.

## Competing interests

None declared.

## Author contributions

MRK and SCT conceptualized the study, MRK collected and curated data, performed analyses, developed methodology and software. SCT provided supervision, secured funding, offered conceptual guidance. MRK wrote the first manuscript draft with contributions from SCT, who also reviewed and edited the manuscript.

## Disclaimer

The New Phytologist Foundation remains neutral with regard to jurisdictional claims in maps and in any institutional affiliations.

## Supporting information


**Fig. S1** Study site and greenhouse gas flux measurement setup.
**Fig. S2** Dynamic leaf chamber connected to trace gas analyzers for simultaneous *in situ* measurement of CH_4_, N_2_O, CO_2_, and H_2_O fluxes from intact foliage.
**Fig. S3** Foliar CH_4_ oxidation and N_2_O emission across shade tolerance classes in deciduous and coniferous species.
**Fig. S4** Effects of functional tolerance classes (drought and waterlogging) on foliar CH_4_ oxidation and N_2_O emission across spring and fall seasons.
**Fig. S5** Seasonal variation in foliar greenhouse gas fluxes across tree families.
**Fig. S6** Phylogenetic variation in foliar gas fluxes across temperate tree species.
**Fig. S7** Species‐level comparison of the relative climate impact of foliar CH_4_ uptake and N_2_O emission.
**Table S1** Sequencing quality and read processing summary.
**Table S2** Mean ± SE of foliar CH_4_ oxidation and N_2_O emission across tolerance classes for shade, drought, and waterlogging.
**Table S3** Seasonal and species‐type effects on foliar CO_2_, CH_4_, N_2_O, and H_2_O fluxes, based on linear mixed‐effects models.
**Table S4** Analysis of variance results showing the effects of functional tolerance classes (drought and waterlogging) and season on foliar CH_4_ oxidation and N_2_O emission.
**Table S5** Variance components, SD, and percentage of total variance explained for foliar CH_4_ oxidation and N_2_O fluxes at each hierarchical level.
Please note: Wiley is not responsible for the content or functionality of any Supporting Information supplied by the authors. Any queries (other than missing material) should be directed to the *New Phytologist* Central Office.

## Data Availability

The source data used to generate all graphs and charts presented in this study are publicly available via the Scholars Portal Dataverse repository at: doi: 10.5683/SP3/LSD4IH.
