# Supplementary material for: Species‐level controls of foliar methane and nitrous oxide fluxes: roles of traits and microbes in temperate trees
Source: New Phytol. 2026 Jul 13;251(6):3221–37. doi: 10.1111/nph.71433 (PMC13491251; doi:10.1111/nph.71433)
Supplement: Supplementary file 1 [file NPH-251-3221-s001.pdf]

New Phytologist Supporting Information

Article title: Species-level controls of foliar methane and nitrous oxide fluxes: roles of traits and microbes in temperate trees

Authors: Md Rezaul Karim, Sean C. Thomas

Article acceptance date: 27 June 2026

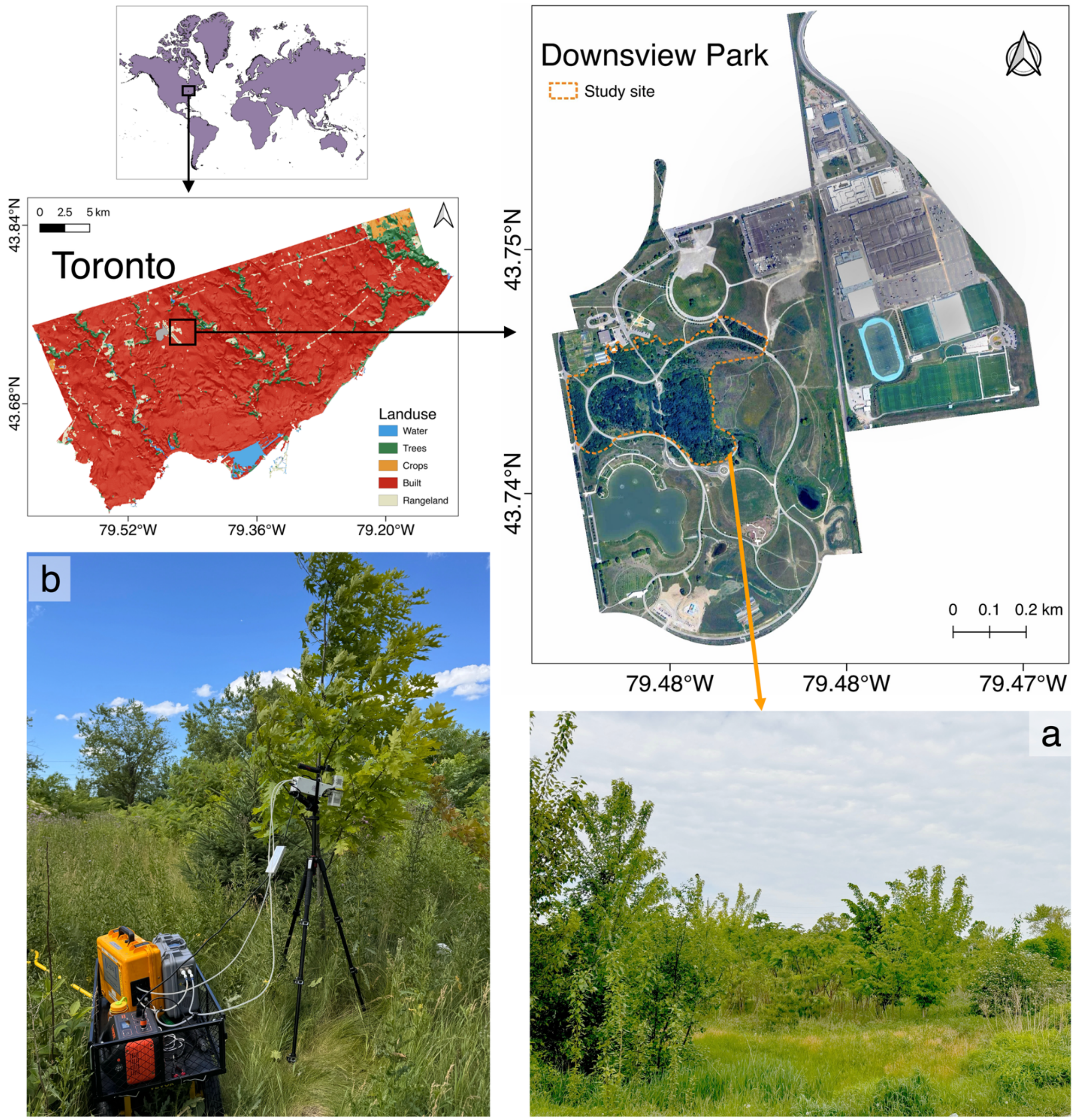

Figure S1: Study site and greenhouse gas (GHG) flux measurement setup. The top panels show the study location in a global context (top left), land cover distribution in Toronto, Ontario, Canada (center left), and the specific boundaries of Downsview Park (right). Bottom panels illustrate (a) a general site view within Downsview Park and (b) the deployment of dynamic leaf chambers on trees for in-situ GHG flux measurements.

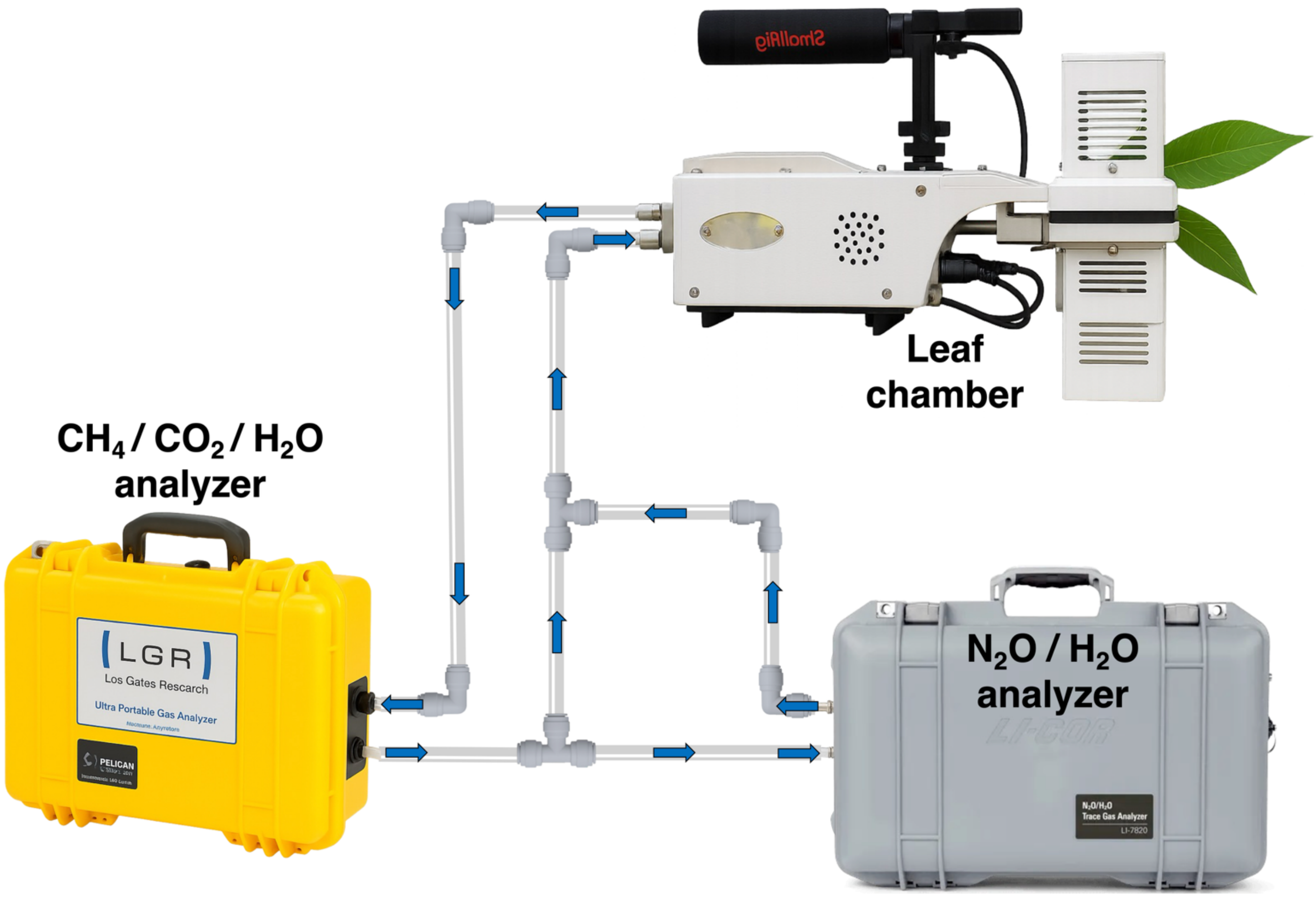

Figure S2: Dynamic leaf chamber connected to trace gas analyzers for simultaneous in situ measurement of CH_4, N_2O, CO_2, and H_2O fluxes from intact foliage.

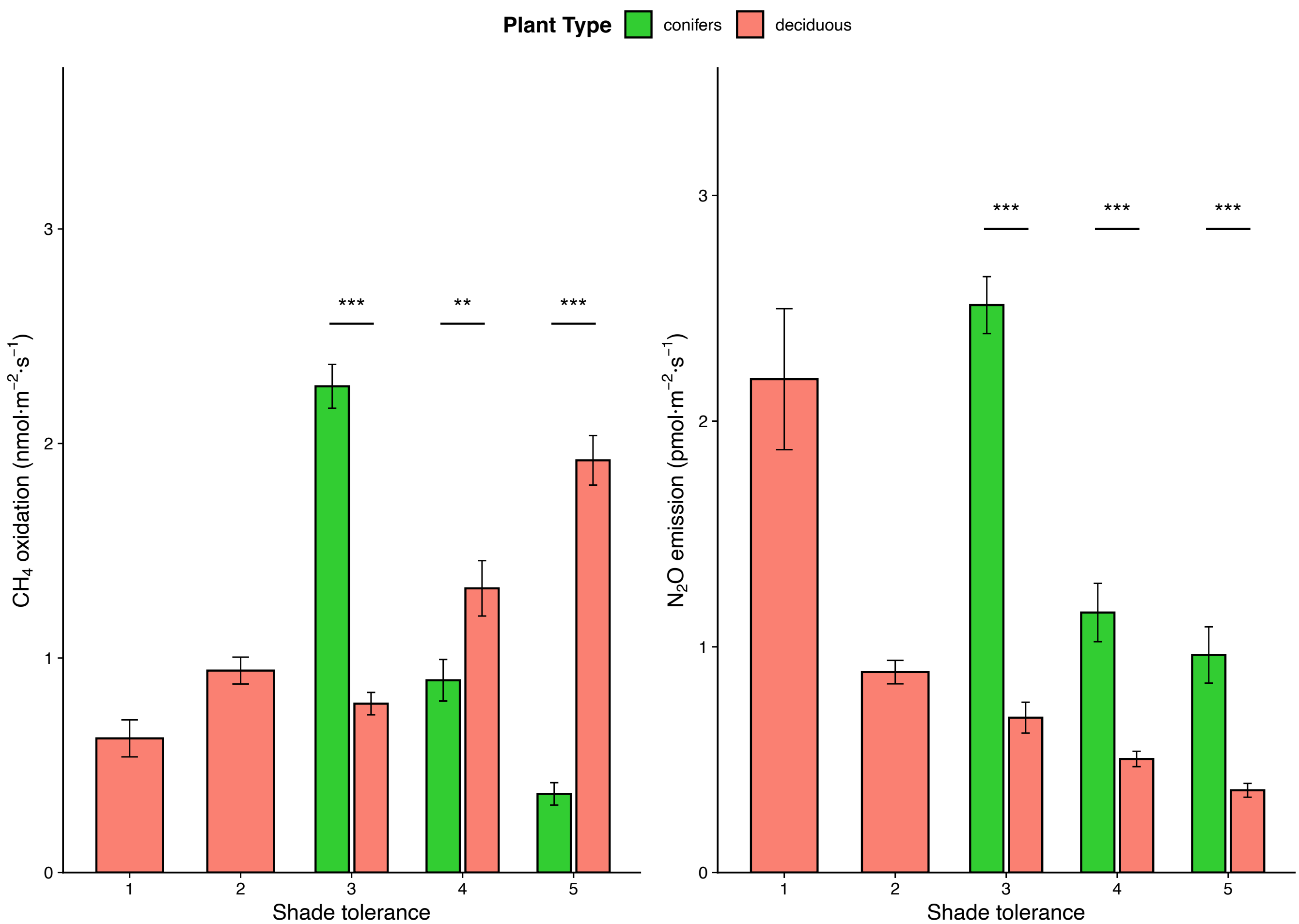

Figure S3: Foliar CH_4 oxidation and N_2O emission across shade-tolerance classes in deciduous and coniferous species. Mean (± SE) foliar CH_4 oxidation (left) and N_2O emission (right) across shade-tolerance classes (1–5) for deciduous and coniferous trees. Bars represent species grouped by plant functional type, with significance indicators denoting pairwise differences between deciduous and coniferous species within each shade-tolerance class (t-tests; $p < 0.05$).

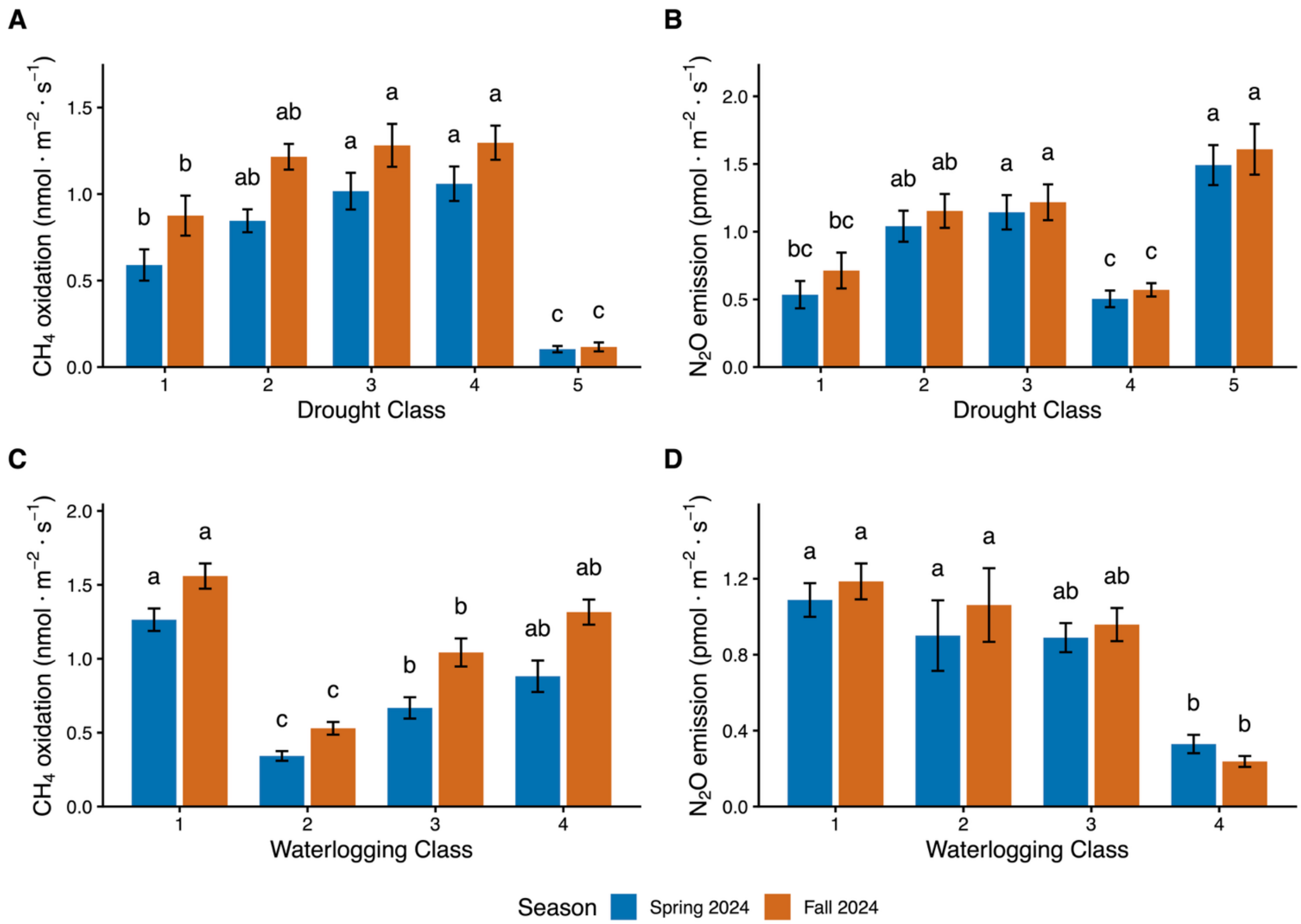

Figure S4: Effects of functional tolerance classes (drought and waterlogging) on foliar CH_4 oxidation and N_2O emission across spring and fall seasons. Bars represent mean fluxes (± SE) for each tolerance class, with letters indicating significant differences based on Tukey's HSD tests ($p < 0.05$). Increasing values along tolerance classes represent transitions from less to more stress-tolerant species.

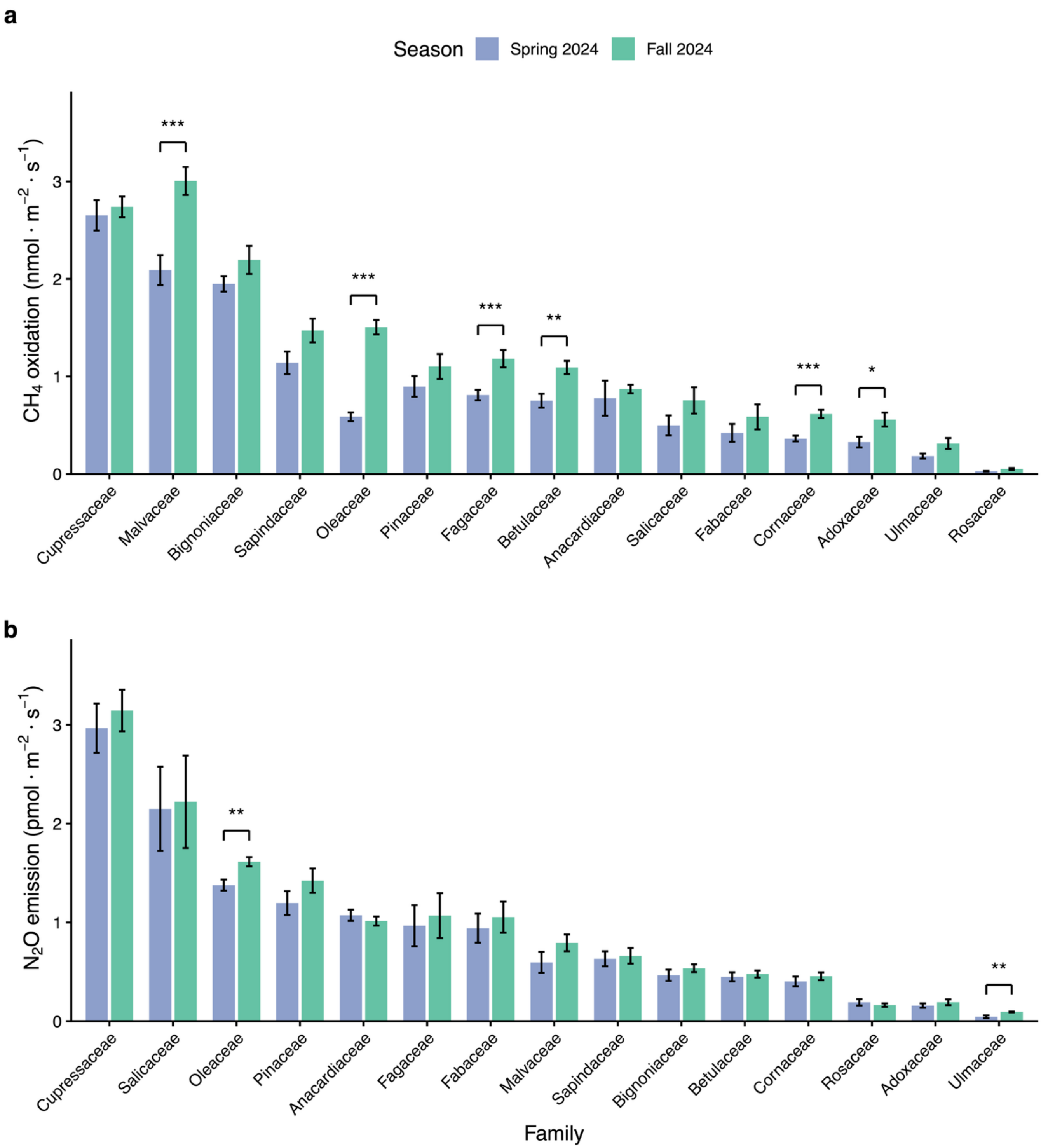

Figure S5: Seasonal variation in foliar greenhouse gas fluxes across tree families. (a) Mean (± SE) rates of CH_4 oxidation (nmol·m^{-2}·s^{-1}) and (b) mean (± SE) rates of N_2O emission (pmol·m^{-2}·s^{-1}) measured on leaves during spring (blue) and fall (green) 2024 across major tree families.

Families are ordered alphabetically; asterisks denote significant seasonal differences (*$p < 0.05$, **$p < 0.01$, ***$p < 0.001$).

a.

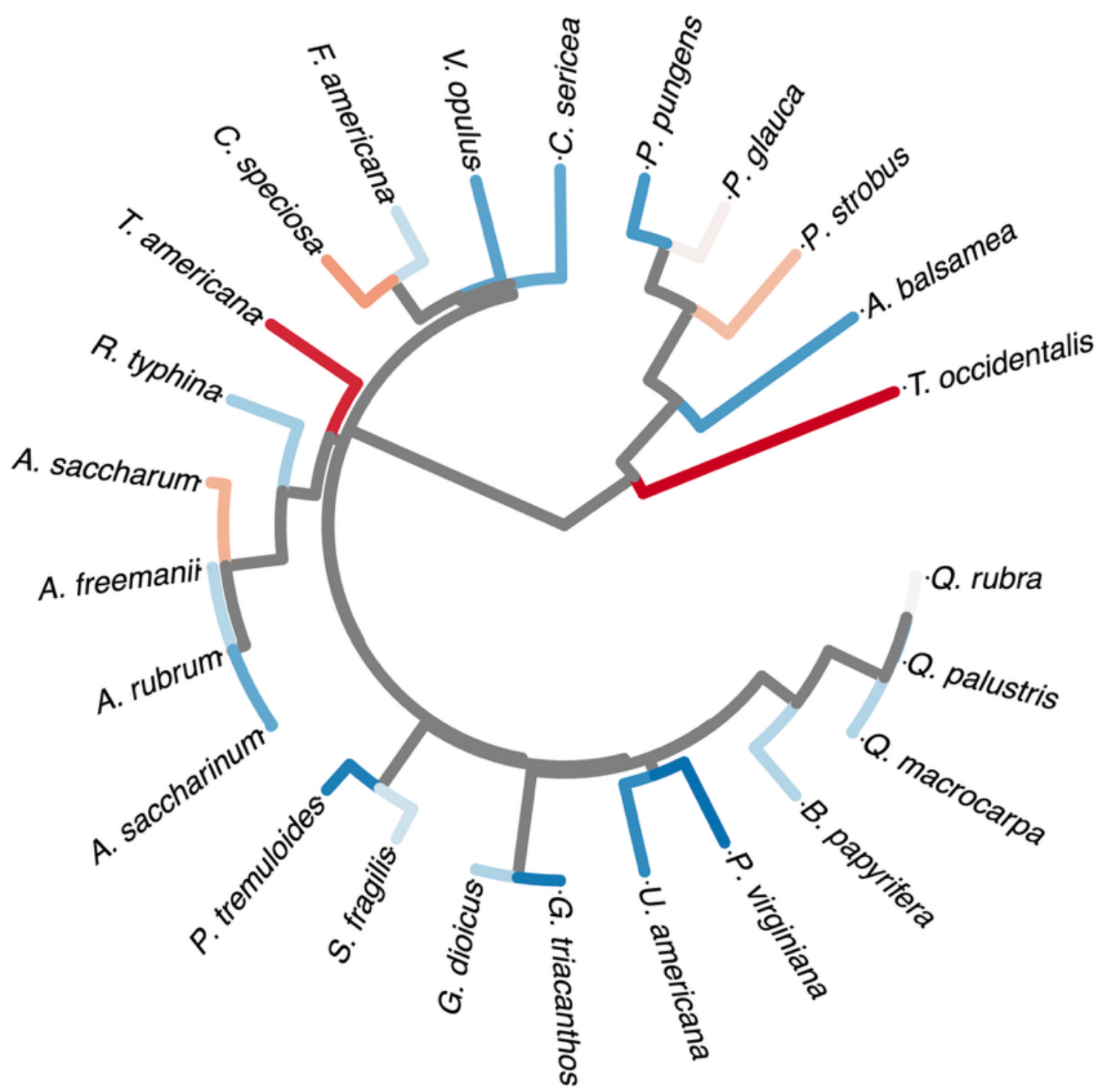
F. americana
V. opulus
C. sericea
P. pungens
P. glauca
P. strobus
A. balsamea
T. occidentalis
C. speciosa
T. americana
R. typhina
A. saccharum
A. freemanii
A. rubrum
A. saccharinum
P. tremuloides
S. fragilis
G. dioicus
G. triacanthos
U. americana
P. virginiana
B. papyrifera
Q. macrocarpa
Q. palustris
Q. rubra
CH4 Oxidation (nmol · m−2 · s−1)
0.5 1.0 1.5 2.0 2.5

b.

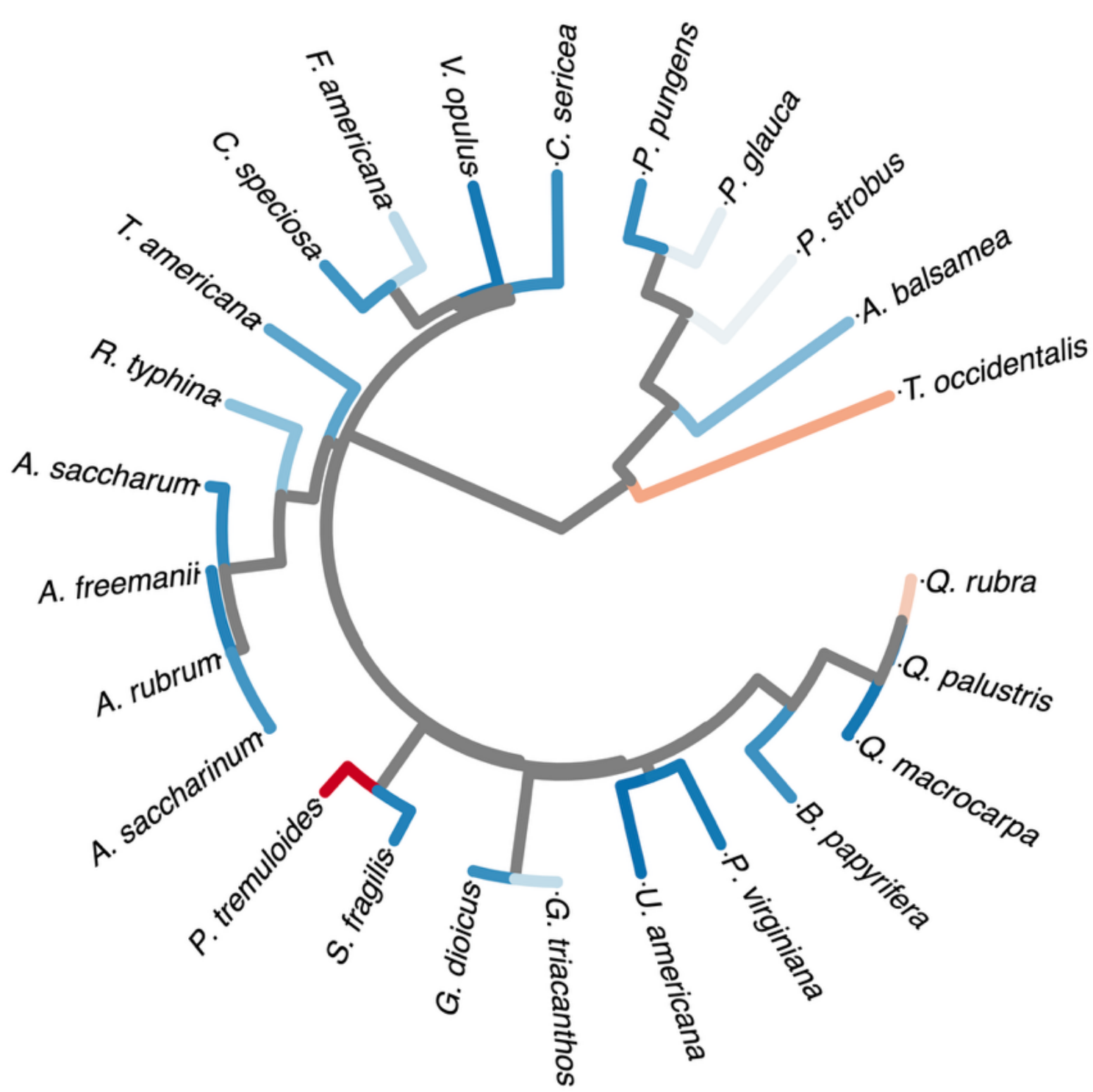
F. americana
V. opulus
C. sericea
P. pungens
P. glauca
P. strobus
A. balsamea
T. occidentalis
C. speciosa
T. americana
R. typhina
A. saccharum
A. freemanii
A. rubrum
A. saccharinum
P. tremuloides
S. fragilis
G. dioicus
G. triacanthos
U. americana
P. virginiana
B. papyrifera
Q. macrocarpa
Q. palustris
Q. rubra
N2O emission (pmol · m−2 · s−1)
1 2 3 4

Figure S6: Phylogenetic variation in foliar gas fluxes across temperate tree species. (a) Leaf CH_4 oxidation and (b) leaf N_2O emission. Tip colors on the phylogenetic tree represent the magnitude of each flux, mapped continuously along a gradient.

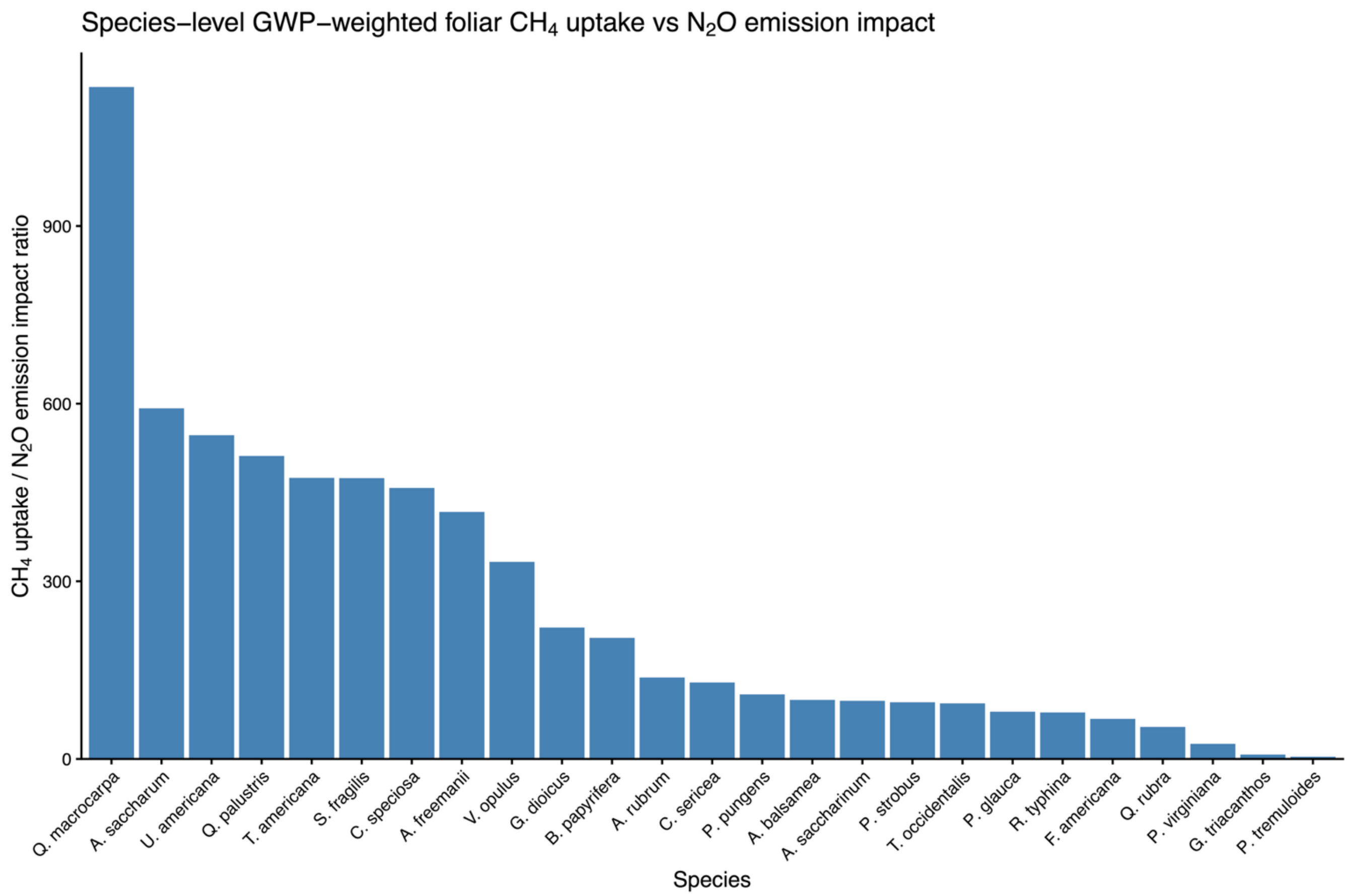

Figure S7: Species-level comparison of the relative climate impact of foliar CH_4 uptake and N_2O emission. Bars represent the mean CH_4-to-N_2O impact ratio (GWP-weighted fluxes) for each species, ordered from highest to lowest. Values >1 indicate that foliar CH_4 oxidation dominates the net climate forcing compared with N_2O emission.

Table S1

Sequencing quality and read processing summary

Amplicon sequence quality and read retention were assessed using DADA2 v1.22 for both the 16S rRNA and pmoA datasets. The workflow included primer trimming, filtering, error correction, merging, and chimera removal. Read retention rates were calculated as the proportion of non-chimeric reads relative to the raw input.

Across all samples, 16S rRNA libraries showed consistently high data quality, with final non-chimeric read counts ranging from 31,242 to 246,502, corresponding to an average recovery of 92.3 ± 1.4%. The pmoA libraries exhibited greater variability, with 1,765–18,937 retained reads (20–73% recovery), reflecting expected differences in target gene abundance among species. Basswood (*Tilia americana*; samples 1–3) generally produced higher read depth and retention compared with Chokecherry (*Prunus virginiana*; samples 5–7), consistent with their distinct methanotrophic profiles.

Table S1-1: DADA2 read processing summary for 16S rRNA gene amplicons

Sample	Raw (input)	Filtered	Denoised F	Denoised R	Merged	Non-chimeric	Retained (%)
Tilia-01	206,234	206,234	203,362	202,595	195,159	193,088	93.6
Tilia-02	264,731	264,729	260,616	259,637	248,812	246,502	93.1
Tilia-03	45,769	45,769	44,775	44,621	42,599	42,542	93.0
Prunus-01	59,630	59,629	58,565	58,435	55,392	52,900	88.7
Prunus-02	37,948	37,948	36,852	36,710	35,111	34,635	91.3
Prunus-03	39,554	39,530	33,702	33,469	31,780	31,242	89.3

Table S1-2: DADA2 read processing summary for pmoA gene amplicons.

Sample	Raw (reads.in)	Filtered	Denoised	Non-chimeric	Retained (%)
Tilia-01	25,927	25,686	19,189	18,937	73.0
Tilia-02	8,398	8,226	3,467	3,467	41.3
Tilia-03	8,263	7,996	1,780	1,765	21.4
Prunus-01	13,672	13,149	116	116	0.9
Prunus-02	12,495	12,090	66	66	0.5
Prunus-03	10,550	10,167	95	95	0.9

In summary, the DADA2 pipeline achieved high-quality recovery for all 16S libraries and variable—but expected—retention for pmoA, confirming the robustness of sequence processing and supporting subsequent community composition analyses.

Table S2: Mean ± standard error (SE) of foliar CH_4 oxidation ($nmol \cdot m^{-2} \cdot s^{-1}$) and N_2O emission ($pmol \cdot m^{-2} \cdot s^{-1}$) across tolerance classes for shade, drought, and waterlogging. Values are shown separately for spring and fall 2024.

Tolerance type	Response	Tolerance class	Spring 2024	Fall 2024
Shade tolerance	CH_4 oxidation ($nmol \cdot m^{-2} \cdot s^{-1}$)	1	0.497 ± 0.102	0.754 ± 0.135
		2	0.787 ± 0.081	1.10 ± 0.0914
		3	0.945 ± 0.084	1.22 ± 0.0903
		4	0.971 ± 0.103	1.34 ± 0.141
		5	1.05 ± 0.193	1.24 ± 0.204
	N_2O emission ($pmol \cdot m^{-2} \cdot s^{-1}$)	1	2.15 ± 0.426	2.22 ± 0.467
		2	0.863 ± 0.068	0.913 ± 0.078
		3	0.982 ± 0.108	1.12 ± 0.116
		4	0.742 ± 0.093	0.784 ± 0.088
		5	0.559 ± 0.099	0.769 ± 0.122
Drought tolerance	CH_4 oxidation ($nmol \cdot m^{-2} \cdot s^{-1}$)	1	0.590 ± 0.091	0.875 ± 0.116
		2	0.846 ± 0.066	1.22 ± 0.074
		3	1.02 ± 0.106	1.28 ± 0.124
		4	1.06 ± 0.099	1.30 ± 0.098
		5	0.104 ± 0.018	0.116 ± 0.025
	N_2O emission ($pmol \cdot m^{-2} \cdot s^{-1}$)	1	0.535 ± 0.101	0.713 ± 0.132
		2	1.04 ± 0.115	1.15 ± 0.125
		3	1.14 ± 0.127	1.22 ± 0.132
		4	0.504 ± 0.062	0.570 ± 0.049
		5	1.49 ± 0.147	1.61 ± 0.187
Waterlogging tolerance		1	1.26 ± 0.076	1.56 ± 0.085

	CH_4 oxidation ($nmol \cdot m^{-2} \cdot s^{-1}$)	2	0.342 ± 0.032	0.529 ± 0.043
		3	0.668 ± 0.072	1.04 ± 0.094
		4	0.882 ± 0.107	1.32 ± 0.085
	N_2O emission ($pmol \cdot m^{-2} \cdot s^{-1}$)	1	1.09 ± 0.088	1.19 ± 0.094
		2	0.901 ± 0.186	1.06 ± 0.194
		3	0.890 ± 0.076	0.958 ± 0.087
		4	0.329 ± 0.048	0.238 ± 0.028

Table S3: Seasonal and species-type effects on foliar CO_2, CH_4, N_2O, and H_2O fluxes, based on linear mixed-effects models. ANOVA statistics (df, F-value, and p-value) indicate the significance of main and interactive effects between species type (deciduous vs. coniferous) and season (spring 2024 vs. fall 2024).

**Response variable**	**Fixed effect**	**Sum Sq**	**Mean Sq**	**Num DF**	**DenDF**	**F value**	**P value**
CH_4 oxidation ($nmol \cdot m^{-2} \cdot s^{-1}$)	Type	1.0766	1.0766	1	248.21	10.3175	0.0014**
	Season	4.7561	4.7561	1	248.77	45.5781	<0.001***
	Type×Season	0.4666	0.4666	1	248.77	4.4712	0.0354*
N_2O emission ($pmol \cdot m^{-2} \cdot s^{-1}$)	Type	2.21027	2.2102	1	248.21	26.1668	<0.001***
	Season	1.74155	1.7415	1	248.49	20.6178	<0.001***
	Type×Season	0.50712	0.5071	1	248.49	6.0037	0.0149*
H_2O flux ($mmol \cdot m^{-2} \cdot s^{-1}$)	Type	3.6772	3.6772	1	248.07	6.1715	0.0136*
	Season	0.8729	0.8729	1	249.03	1.4650	0.2272
	Type×Season	0.7114	0.7114	1	249.03	1.1940	0.2755
CO_2 uptake ($\mu mol \cdot m^{-2} \cdot s^{-1}$)	Type	154.926	154.926	1	248.29	32.9452	<0.001***
	Season	2.176	2.176	1	248.87	0.4627	0.4969
	Type×Season	15.956	15.956	1	248.87	3.3931	0.0666

Table S4: Analysis of variance (ANOVA) results showing the effects of functional tolerance classes (drought and waterlogging) and season on foliar CH_4 oxidation and N_2O emission.

Factor type	Response variable	Source of variation	Df	Sum Sq	Mean Sq	F value	Pr(>F)
Drought tolerance	CH_4 oxidation	Drought class	4	24.49	6.123	9.817	**<0.001**
		Season	1	10.80	10.798	17.312	**<0.001**
		Drought × Season	4	0.78	0.196	0.314	0.868
	N_2O emission	Drought class	4	35.8	8.961	8.402	**<0.001**
		Season	1	1.2	1.211	1.136	0.287
		Drought × Season	4	0.1	0.030	0.028	0.998
Waterlogging tolerance	CH_4 oxidation	Waterlogging class	3	81.09	27.030	53.374	**<0.001**
		Season	1	10.80	10.798	21.322	**<0.001**
		Waterlogging × Season	3	0.64	0.213	0.421	0.738
	N_2O emission	Waterlogging class	3	15.4	5.134	4.653	**0.003**
		Season	1	1.2	1.211	1.098	0.295
		Waterlogging × Season	3	0.3	0.108	0.098	0.961

Table S5: Variance components, standard deviations (SD), and percentage of total variance explained for foliar CH_4 oxidation and N_2O fluxes at each hierarchical level.

Leaf flux	Hierarchical level	Variance	SD	Variance explained (%)
CH_4	Family	8.70×10^{-10}	2.95×10^{-5}	≈0.00
	Species	0.550	0.742	80.0
	Tree (individual)	0.011	0.105	1.6
	Leaf	2.68×10^{-10}	1.64×10^{-5}	≈0.00
	Residual	0.124	0.352	18.0
N_2O	Family	2.69×10^{-8}	1.64×10^{-4}	≈0.00
	Species	1.028	1.014	91.0
	Tree (individual)	5.57×10^{-3}	0.075	0.49
	Leaf	6.02×10^{-3}	0.078	0.53
	Residual	0.092	0.303	8.1
